# Defective efferocytosis links autonomic dysfunction and atrial fibrillation: multi-omics integration and *in vivo* validation

**DOI:** 10.3389/fimmu.2026.1818859

**Published:** 2026-05-22

**Authors:** Xiaohan Zhang, Yihang Du, Shuqing Shi, Yuandong Liu, Zelin Ye, Ruoning Chai, Meiyu Feng, Jiaran Li, Limei Zhang, Qingqiao Song, Bai Du, Yuanhui Hu

**Affiliations:** 1 Department of Cardiovascular, Guang’anmen Hospital, China Academy of Chinese Medical Sciences, Beijing, China; 2 Department of Internal Medicine, Guang’anmen Hospital, China Academy of Chinese Medical Sciences, Beijing, China; 3 State Key Laboratory of Cardiovascular Disease, Fuwai Hospital, National Center for Cardiovascular Diseases, Chinese Academy of Medical Sciences & Peking Union Medical College, Beijing, China; 4 Shandong First Medical University, Jinan, China

**Keywords:** Atrial fibrillation, autonomic dysfunction, CD47, efferocytosis, valsartan

## Abstract

**Introduction:**

Atrial fibrillation (AF) is the most common and clinically significant arrhythmia requiring treatment. Although an imbalance between the sympathetic and vagal branches of autonomic nervous system (ANS) is known to trigger and sustain AF, the underlying mechanisms remain incompletely understood. Furthermore, there are yet no clinically authorized antiarrhythmic drugs currently targeting the modulation of ANS.

**Methods:**

Targeted LC–MS/MS profiling of plasma 39 neurotransmitters was conducted in a clinical cohort. To identify shared mechanisms, atrial transcriptomes and a sympathetic neuron activation dataset were integrated with DEGs, WGCNA, enrichment, immune infiltration, and machine-learning feature selection, and in silico docking was performed. For *in vivo* validation, an Ang II–infused mouse model with valsartan intervention was utilized, including transesophageal burst pacing, immunostaining, immunoblotting, and efferocytosis quantification.

**Results:**

Plasma from AF patients exhibited an autonomic-imbalance signature (upregulated monoaminergic and downregulated cholinergic neurotransmitters). Shared ANS–AF bioinformatic analysis highlighted hub genes as CDKN2D, FYTTD1, LRR1, and POPDC3. Immune analyses pinpointed CD47-mediated efferocytosis as a crucial link. *In vivo*, Ang II induced autonomic remodeling, Ca²⁺-handling suppression, increased apoptosis with CD47/SIRPα upregulation, impaired efferocytosis, and heightened inflammatory signaling; valsartan partially reversed these changes.

**Discussion:**

Multi-omics and *in vivo* evidence suggests an ANS–immune–atrial remodeling axis in which CD47–SIRPα–dependent efferocytosis blockade is highly associated with atrial inflammation and AF susceptibility, with valsartan acting as a clinically relevant modulator of this pathway.

## Graphical Abstract

**Figure:**
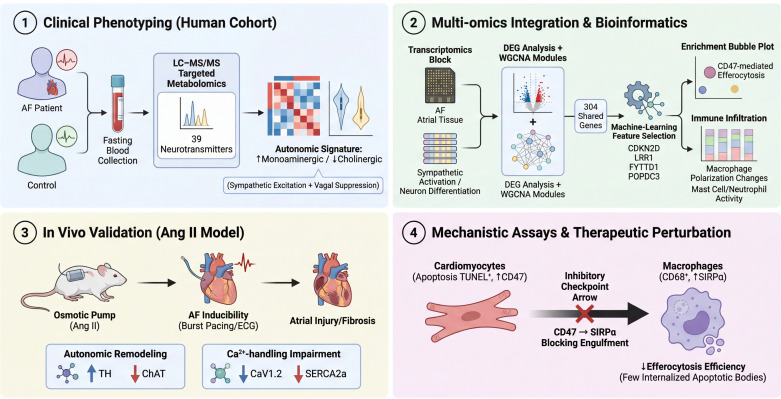

## 1 Introduction

Atrial fibrillation (AF), the common precondition of stroke and heart failure, is characterized by disordered atrial electrical activation and contraction, manifesting as palpitations, chest tightness, impaired exercise capacity, and anxiety (1–3). The globally rising prevalence contributes to increased disability and mortality, imposing a substantial burden on healthcare systems. The autonomic nervous system (ANS) regulates cardiac electrical conduction via the sympathetic and vagus nerves (4). Common AF risk factors, such as obstructive sleep apnea (OSA), bulimia, alcohol consumption, and anxiety or depressive states, may cause sympathetic or vagal dysregulation, resulting in ANS-dysfunction-related AF (5–7). As proposed by Levy’s concept of accentuated antagonism, the basic properties of the vagus and sympathetic nerves often conceptualized as “yin and yang”, yet their intricate interactions are anatomically supported by mixed fibers within both systems (8–10). Crucially, autonomic dysregulation triggers aberrant rapid discharges that precede AF, specifically, sympathetic hyperactivity surges seconds before paroxysmal AF (PAF) onset and remains elevated post-episode (11, 12). Compared with asymptomatic AF, symptomatic PAF exhibits higher sympathetic excitation, with symptom severity correlated with sympathetic activity rather than heart rate changes. Moreover, symptomatic PAF exhibits higher sympathetic tone compared to asymptomatic AF, with symptom severity correlating closely with sympathetic activity rather than isolated heart rate changes (13). Sympathetic hyperactivity stimulates β_1_-adrenoceptors, increasing cardiomyocyte automaticity and accelerating calcium influx (14). The intracellular calcium overload shortens the atrial effective refractory period (AERP) and generates a pro-arrhythmic substrate (15). The vicious loop between AF and autonomic remodeling is a core aspect of the classic “AF begets AF” paradigm (16).

Inflammation, particularly immune infiltration in the epicardial adipose tissue (EAT) microenvironment, drives AF pathogenesis (17, 18). Atrial tissues from AF patients exhibit notable macrophage accumulation, which activates fibroblasts and drives structural remodeling (19). Importantly, the ANS actively modulates this local immune response. The sympathetic nervous amplifies pro-inflammatory cytokine effects in afferent nerves, potentiating neuroinflammatory AF, while the vagus nerve provides anti-inflammatory neural circuits (20, 21). These observations support the hypothesis that chronic low-grade atrial inflammation serves as an effect amplifier mediating autonomic dysregulation-induced AF promotion (22).

Currently, AF management faces significant therapeutic bottlenecks. Notably, no clinically approved antiarrhythmic drugs specifically target ANS modulation, and the multi-pharmacologic effects of existing agents require further mechanistic exploration. This study combined transcriptomic datasets of AF and sympathetic neurons activation to identify overlapped differentially expressed genes (DEGs) and elucidates the biological pathways underlying ANS-dysfunction-related AF. Machine learning methods were also integrated to screen hub biomarkers. Then, the role of inflammation as the potential contributor is jointly delineated by immune infiltration analysis. Finally, *in-vivo* validation were employed to explore whether the progression of autonomic remodeling to a pro-arrhythmic substrate is associated with impaired efferocytosis and resultant pro-inflammatory effects. These insights propose a novel strategy targeting ANS for AF management and elucidates the novel antiarrhythmic targets of angiotensin II receptor blockers (ARB) valsartan.

## 2 Materials and methods

### 2.1 Cohort participants and sample collection

This cross-sectional study enrolled patients from Guang’anmen Hospital, China Academy of Chinese Medical Sciences, between June and December 2022. All participants provided written informed consent, and the study protocol was approved by the Ethics Committee of Guang’anmen Hospital (No.2022-221-KY). Patients with PAF and persistent AF (PerAF) comprised the study cohort, which was compared with age-matched healthy controls from the Cadre Health Care Department. The diagnostic criteria for AF are conformed in accordance with the European Society of Cardiology (ESC) guidelines (23, 24).

Peripheral blood samples were collected from fasting (≥8 h) during a standardized morning phlebotomy window (07:00–09:00) before breakfast and before routine morning medication, using EDTA anticoagulant tubes. After gentle mixing, samples were centrifuged at 800×g for 20 minutes at 4 °C. Plasma was aliquoted into EP tubes, and stored at −80 °C.

### 2.2 Targeted neurotransmitter metabolomics

A standard curve was prepared using serial dilutions of a 39-neurotransmitter reference standard mixture. Each 20 μL aliquot of sample or standard was treated with 60 μL of pre-cooled 50% methanol, vortexed for 5 min, precipitated at −20 °C for 4 h, and centrifuged at 20,000×g for 15 min at 4 °C. Then, 20 μL supernatant was subjected to derivatization using 20 μL of 200 mM 3-NPH and 20 μL of 120 mM EDC–6% pyridine at 25 °C for 30 min. After centrifugation, the supernatant was analyzed by LC–MS/MS.

Analysis was performed on a Waters I-Class AB Sciex 6500 system equipped with a BEH C18 column (1.7 μm, 2.1 × 100 mm). The mobile phases consisted of 0.1% formic acid in water (A) and methanol (B), with a flow rate of 0.35 mL/min using the following gradient: 2% B at 0–2 min, increasing to 20–80% B from 2.5 to 15 min. Quantification was based on peak area integration against the standard curve using Analyst software.

### 2.3 Transcriptomic datasets retrieval and DEGs analysis

Transcriptomic data for AF and ANS were obtained from the NCBI Gene Expression Omnibus (GEO) database (http://www.ncbi.nlm.nih.gov/geo). AF datasets, including GSE41177, GSE115574, and GSE79776, were sourced from atrial tissues collected during valve surgery (25–27). After merging these datasets, 49 sinus rhythm (SR) samples served as controls and 74 AF samples as the disease cohort. Surrogate variables were generated using the “sva” R package to alleviate batch effects across datasets for subsequent high-dimensional analysis. Expression profile of autonomic neuron was obtained from GSE80689, originating from human pluripotent stem cell-derived sympathetic neurons that modulate cardiomyocyte electrophysiology (28). Here, 3 undifferentiated samples served as controls, while 5 samples at the sympathetic neuron precursor stage constituted the disease group. Both expression matrices underwent probe-to-gene symbol conversion, missing value imputation, and normalization.

DEGs identification was conducted using the “LIMMA” R package in expression arrays of both AF cohorts and sympathetic neuron activation. To ensure analytical rigor, stratified statistical thresholds were implemented, with AF DEGs required |log_2_FC| ≥ 0.8 and *P* < 0.05, and sympathetic activation DEGs required |log_2_FC| ≥ 1 and *P* < 0.05. The “pheatmap” and “ggplot2” packages were employed for visualization. Ultimately, intersecting DEGs from AF and sympathetic neuron activation were defined as shared genes contributing to ANS dysfunction-related AF.

### 2.4 Weight gene co-expression network construction

Gene expression profiles from both disease datasets were carefully filtrated, retaining the top 10,000 highest-expressed genes followed by selection of the 5,000 most variable genes (29). The optimal soft thresholding power (β-value) was determined via stepwise selection to achieve scale-free network topology (30). Gene modules were identified using the dynamic tree-cutting algorithm, with subsequent merger of small modules (<30 genes) to enhance biological robustness. Pearson correlation coefficients were used to quantify module-disease trait correlations (P<0.01). Combined with DEGs, overlapping genes in these disease-associated modules were identified as potential co-regulated genes with common pathology.

### 2.5 Machine learning algorithms for hub genes screening

Machine learning algorithms enable multidimensional analysis of omics data, significantly enhancing the reliability of subsequent analyzes (31). LASSO regression was performed using the “glmnet” R package (family = binomial), and key feature genes were found by optimizing the regularization parameter λ by 10-fold cross-validation (model selection based on λ.min). Subsequently, support vector machine-recursive feature elimination (SVM-RFE) was executed with the “caret” package with a radial basis kernel, where nested 10-fold cross-validation identified suitable feature subsets based on model accuracy evaluation. Random Forest (RF) modeling was conducted using 500 decision trees (ntree = 500) for both AF and ANS arrays, with feature importance assessed by mean decrease Gini metrics. Finally, the predictive power assessment of machine learning-derived logistic regression models employed receiver operating characteristic (ROC) analysis (“pROC” package), calculating area under the curve (AUC) values with ROC curve generation (32).

### 2.6 Immune infiltration analysis

CIBERSORT analysis was performed on standardized expression matrices using the LM22 signature matrix (22 immune cell subtypes). Using support vector regression, the tool deconvoluted bulk tissue gene expression to estimate immune cell abundances. With 1,000 permutations, compositional heterogeneity was assessed across all samples. Differential immune composition between AF and ANS arrays was evaluated by Wilcoxon rank-sum tests, with mean abundance differences calculated and FDR-adjusted (Benjamini-Hochberg method). Infiltration patterns were visualized, and Pearson correlations were calculated across all 22 immune cells to create interaction matrices, which were then visualized using heatmaps.

### 2.7 Mice and the angiotensin II-induced AF susceptibility model

All mice were on a C57BL/6J background (male, 8 weeks), weighing 22 ± 2g were obtained from Beijing WeiTong LiHua Laboratory Animal Technology Co. for the study. The 30 mice were housed in specific pathogen free (SPF) animal labs with a 12-hour light/dark cycle and a temperature of 25 °C.

To establish models of AF susceptibility, mini-osmotic pump implantation were implemented. Under anesthesia with 1% sodium pentobarbital (intraperitoneal injection, 50mg/kg), osmotic pumps (Alzet ^®^ model #2004, 200 μl, 28 days) containing Ang II or saline (1600ng/kg/min) were inserted subcutaneously (33). All mice were randomly and equally assigned into saline group, Ang II group, and Ang II + valsartan group (30mg/kg/day, 6 weeks) (34). Following 4-week continuous drug infusion via osmotic pressure, echocardiography evaluating cardiac function were performed as previous (35). And after 6-week administration, mice were sacrificed and atrial tissues were harvested, HE and Masson staining were employed to observe cardiomyocyte morphology and fibrosis.

All experimental procedures and animal welfare practices strictly adhered to the Ethical Regulations on the Care and Use of Laboratory Animals of Guang’anmen Hospital to ensure the ethical treatment of the animals (IACUC-GAMH-2024-058).

### 2.8 Transesophageal atrial burst pacing

Mice were anesthetized by inhalation of isoflurane (1.5%–2.0%) and secured in the supine position. Subcutaneous limb electrodes were connected to an electrocardiogram (ECG) recording system to acquire lead II surface baseline ECG stabilization for 3 min. Then, a custom-made transesophageal pacing/recording catheter was introduced orally and advanced to position the electrode at the mid–left atrial level. Proper placement was confirmed by the transesophageal electrogram, in which the atrial A-wave exhibited a positive–negative (qR-shaped) morphology. Once the heart rate was stable, atrial burst pacing was delivered at 2× the capture threshold (basic cycle length, 20 ms; pulse width, 2 ms) for 5 s per train, repeated 10 times (33). AF induction was considered successful when rapid and fragmented atrial electrograms persisted for ≥3 s in the presence of irregular atrioventricular nodal conduction and ventricular rhythm, occurring on at least three occasions. AF duration was defined as the interval from AF onset to spontaneous termination, and AF inducibility was quantified by the number of AF episodes and the cumulative AF duration.

### 2.9 Western blotting

Mouse atrial tissues were minced in a 1.5mL microcentrifuge tube. Lysates were centrifuged and the supernatants were collected. Protein concentration was determined using a BCA assay kit (Beyotime, P0010), and 50 μg total protein per lane was loaded. PVDF Membranes were incubated with primary antibodies diluted in blocking buffer overnight at 4 °C. 1:10000 β-actin (Proteintech, 66009-1-Ig), 1:500 CDKN2D (Proteintech, 10272-2-AP); 1:10000 TH (Abcam, ab137869); 1:1000 ChAT (Abcam, ab178850); 1:1000 SIRPα (CST, 13379T); 1:1000 IL-6 (Abcam, ab290735); 1:500 TNF-α (Proteintech, 17590-1-AP); 1:500 iNOS (Proteintech, 18985-1-AP); 1:1000 IL-1β (Proteintech, 26048-1-AP); 1:1000 p65 (CST, 8242); 1:1000 p-p65 (CST, 3033), and then incubated with 1:1000 HRP-conjugated secondary antibody (Beyotine, A0208). Then, membranes were washed in PBST and reprobed with β-actin as the loading control following the same secondary incubation and ECL detection procedures.

### 2.10 Reverse transcription–quantitative PCR

Total RNA was extracted using TRIzol and dissolved in 50 µL RNase-free water, followed by DNase I treatment to remove genomic DNA and reverse transcription with M-MLV using oligo(dT)18 primers. Transcript levels were quantified by RT–qPCR using TB Green^®^ Premix Ex Taq™ II on a LightCycler 480 system (Roche; 384-well format), with GAPDH as the internal reference. Amplification was performed under standard cycling conditions (95 °C pre-denaturation, 40 cycles of 95 °C/60 °C for fluorescence acquisition). A melt-curve analysis was performed from 65 °C to 95 °C to confirm amplification specificity, and all reactions were run in triplicate. Primer sequences are provided in **Table 1**.

**Table 1:** Primers used for RT-qPCR.

Gene	Forward primer	Reverse primer
GAPDH	GAGCCAAAAGGGTCATCATCT	AGGGGCCATCCACAGTCTTC
CDKN2D	GCCACTGTCTCCAGCCTTACT	CAATAAACCACAAACTGCTCCTC
FYTTD1	TCTCCACTCTTGGTGTTCCTCTGA	AGGCTCATCTACTTCTGTGCTCTG
POPDC3	CCGTGCTCATCTCAGGAAGAATCA	CTCCAAGACACATATCGGCAATCG

### 2.11 Immunofluorescence and laser confocal

Paraffin-embedded tissue sections were deparaffinized in xylene for 5–10 min, rehydrated in absolute ethanol for 5 min, and then passed through graded ethanol to double-distilled water. Antigen retrieval was performed using 1× citrate buffer. The TUNEL working solution was prepared according to the manufacturer’s instructions, and 50 μL was applied to each section followed by incubation at 37 °C for 2–3 hours in the dark, ensuring the sections did not dry out. Sections were blocked with serum from the same host species as the second antibody at 37 °C for 30 min, then incubated with primary antibodies diluted in PBS overnight at 4 °C in a humidified chamber. After washing in PBS three times on a shaker, sections were incubated with the appropriate fluorophore-conjugated secondary antibodies at room temperature for 50 min in the dark. Nuclei were counterstained with DAPI for 10 min at room temperature in the dark, followed by three additional PBS washes. Slides were mounted with an anti-fade mounting medium and imaged using a Nikon upright fluorescence microscope.

For efferocytosis analysis, macrophage-associated apoptotic signals were assessed in randomly selected atrial fields stained for CD68 and TUNEL. TUNEL-positive signals spatially enclosed within or closely overlapping with CD68-positive macrophage areas were defined as macrophage-associated apoptotic signals, whereas clearly separated signals were excluded. Efferocytosis efficiency was calculated as the percentage of CD68-associated TUNEL-positive signals among total TUNEL-positive signals in the analyzed fields.

### 2.12 Statistic analysis

SPSS 26.0 and GraphPad Prism 9.0 software were employed to statistically analyze the measurement data. The mean ± SD was used to show data that had a normal distribution. The T-test was used for two group comparisons, and one-way analysis of variance (ANOVA) was used for multiple comparisons. *P* < 0.05 indicates a statistically significant difference, whereas *P* < 0.01 indicates higher confidence.

## 3 Results

### 3.1 Neurometabolic alterations in patients with AF

**Figure 1:**
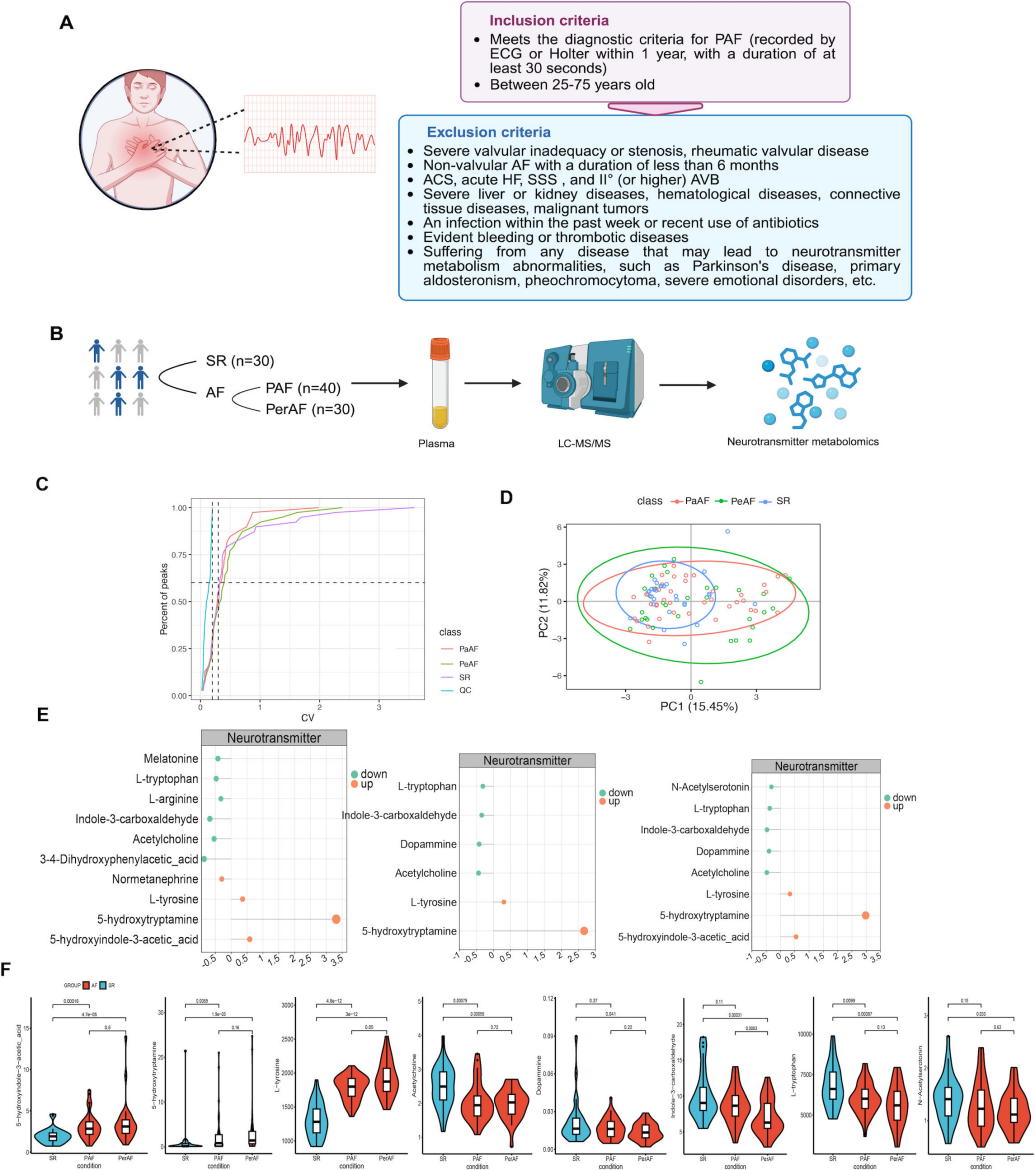
Targeted neurotransmitter metabolomics for AF patients. **(A)** The inclusion and exclusion criteria of AF patients for cross-sectional study. **(B)** Schematics of participant enrollment, plasma collection and targeted metabolites. **(C, D)** Quality control of targeted neurotransmitters metabolites based on CV and PLS-DA methods. **(E)** Differential analysis was performed using independent t-tests. From left to right, the differentially expressed neurotransmitters were identified through comparisons between the PerAF vs SR groups, PAF vs SR groups, and overall AF vs SR groups, respectively (∣FC∣ >1.2 and *P* < 0.05). **(F)** The violin plots visualizing the distribution differences of the upregulated and downregulated neurotransmitters across subgroups within the AF cohort.

According to strict inclusion and exclusion criteria, a total of 100 participants were enrolled in this cohort study, including 70 AF patients (30 with PerAF and 40 with PAF) and 30 participants in the SR group (**Figure 1A**). Baseline clinical characteristics, including the prevalence of hypertension, hyperlipidemia, type 2 diabetes, and heart failure, were well-matched between the cohorts (**Supplementary Table 1**). In contrast, the AF group demonstrated significantly higher levels of homocysteine and high-sensitivity C-reactive protein (hs-CRP), along with lower HDL-C levels. These biochemical differences may correlate to systemic chronic low-grade inflammation and metabolic dysregulation associated with AF.

The neurometabolic profile of AF patients was conducted using targeted metabolomics analysis of 39 neurotransmitters in fasting plasma samples collected under standardized morning conditions (**Figure 1B**). Based on coefficient of variation (CV) and partial least squares-discriminant analysis (PLS-DA), AF patients demonstrated stable and significant differences in neurotransmitter metabolism compared to SR participants (**Figures 1C, D**). Overall, AF patients exhibited dysregulated ANS activity, characterized by upregulated monoamine neurotransmitters and downregulated cholinergic neurotransmitters (**Figure 1E**). Specifically, levels of 5-hydroxyindole-3-acetic acid, 5-hydroxytryptamine, and L-tyrosine were elevated, whereas acetylcholine, dopamine, indole-3-carboxaldehyde, L-tryptophan, and N-acetylserotonin were reduced in the AF cohort (**Figure 1F**). Notably, these circulating neurotransmitters should be interpreted as systemic peripheral markers rather than direct measurements of intracardiac autonomic nerve activity. Therefore, the plasma profile might suggest sympathetic excitation with relative vagal suppression in the atrial microenvironment.

### 3.2 Identification for shared genes of ANS dysregulation-related AF

**Figure 2:**
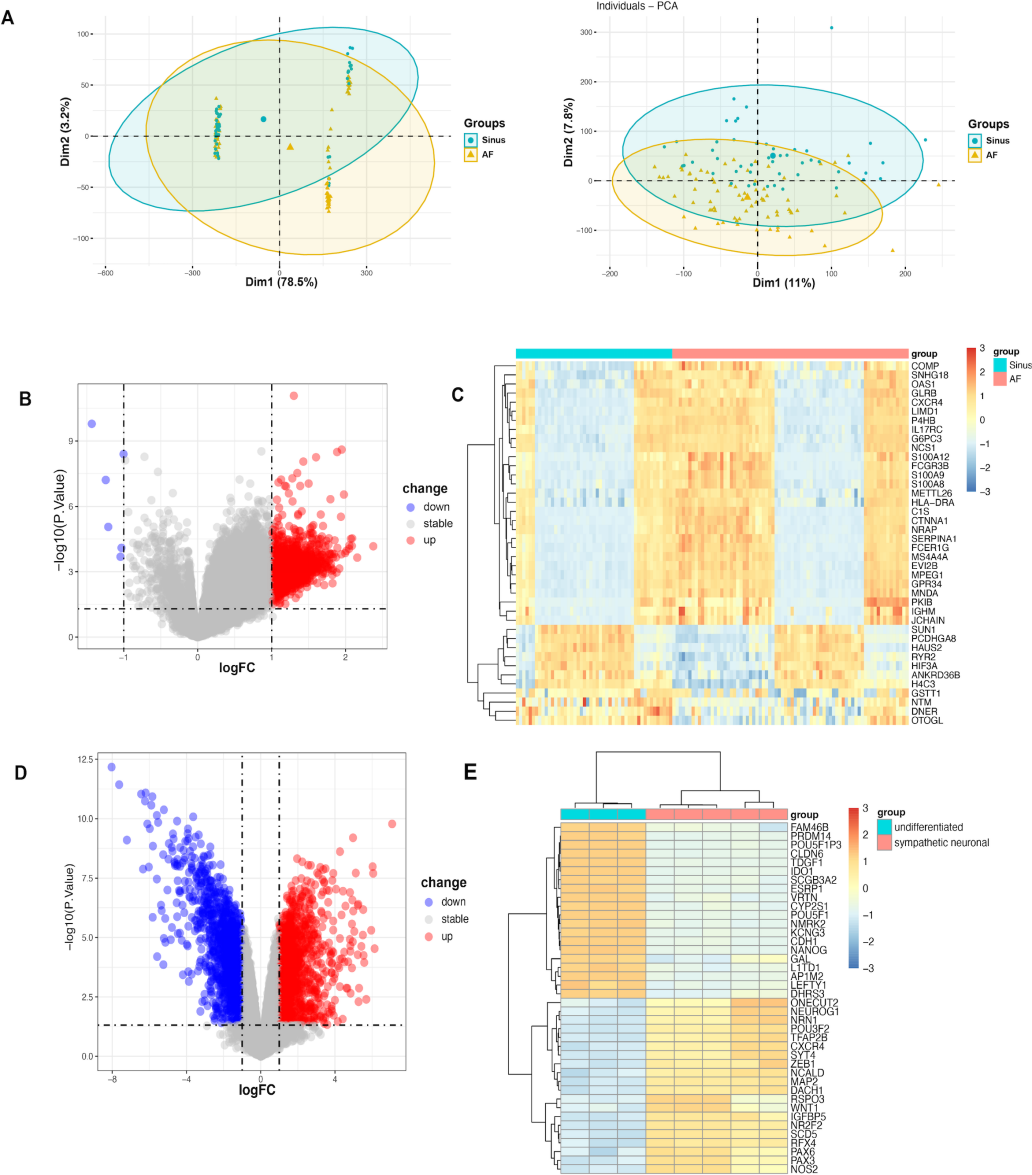
Differential expression gene analysis of AF and ANS datasets. **(A)** PCA plots of expression patterns in 3 AF datasets before and after removing batch effects. **(B)** Volcano plot of DEGs in AF. **(C)** Heatmap of the top 40 DEGs with the highest ∣logFC∣ in the AF datasets. **(D)** Volcano plot of DEGs in sympathetic neuronal differentiation. **(E)** Heatmap of the top 40 DEGs with the highest ∣logFC∣ in sympathetic neuronal differentiation.

Following batch effect removal, the integrated analysis of AF datasets (GSE41177, GSE115574, and GSE79768) revealed expression profiles containing 20824 genes (**Figure 2A**). To ensure the robust and reliable identification of DEGs, thresholds of |log_2_FC| ≥ 0.8 and adjusted *P* < 0.05 were applied and 2205 AF DEGs were identified, among which 11 genes were downregulated and 2194 were upregulated (**Figures 2B, C**). In the sympathetic neuron activation dataset (GSE80689) containing 19431 genes expression, 2616 DEGs were identified, including 1255 downregulated and 1361 upregulated genes (**Figures 2D, E**). A total of 297 overlapping DEGs were acquired between the two conditions, which may serve as core shared genes of ANS dysregulation-related AF for further functional investigation.

**Figure 3:**
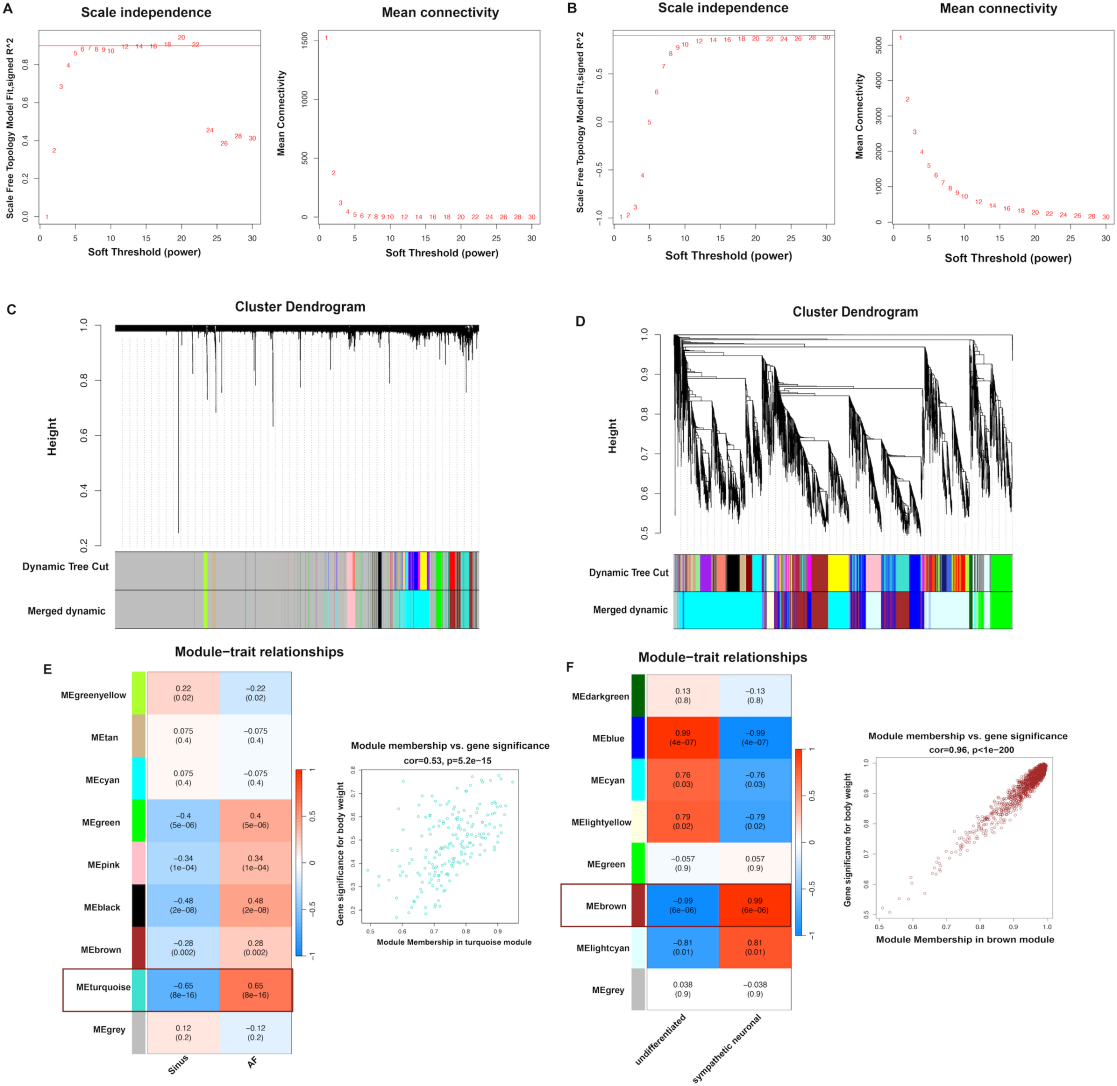
The construction of WGCNA module. **(A)** Scale-free topology fit index analysis (left panel) and gene connectivity analysis (right panel) for AF. **(B)** Scale-free topology fit index analysis (left panel) and gene connectivity analysis (right panel) for sympathetic nerve activation. **(C)** Clustering dendrogram of highly connected genes in AF. **(D)** Clustering dendrogram of highly connected genes in sympathetic nerve activation. **(E–F)** Correlation heatmap between key modules and traits in AF and sympathetic nerve activation.

Meanwhile, to map the correlation between the disease phenotype and core genes, a WGCNA was performed on the expression profiles of the AF and sympathetic neuron activation datasets. Based on scale-free topology, a soft-thresholding power of 20 was selected to construct the network (fit index R² > 0.85), and a soft threshold of 20 was chosen for both datasets (**Figures 3A, B**). Subsequently, an adjacency function was applied to generate a topological overlap matrix (TOM), and hierarchical clustering was constructed, where the height of the dendrogram represents the similarity of gene clustering (**Figures 3C, D**). In the clustering for AF, 14 modules were initially obtained and consolidated into 8 modules, and the turquoise module was ultimately chosen for AF with the highest Pearson correlation scores (**Figure 3E**). For sympathetic nerve activation dataset, 23 modules were initially identified and integrated into 8 modules, and the brown module was finally selected (**Figure 3F**). The intersection of these two modules yielded 9 overlapped genes: TMEM50B, ARL2BP, BOLA3, CD47, ECI2, FYTTD1, LRR1, RPS3, and CUEDC2.

**Figure 4:**
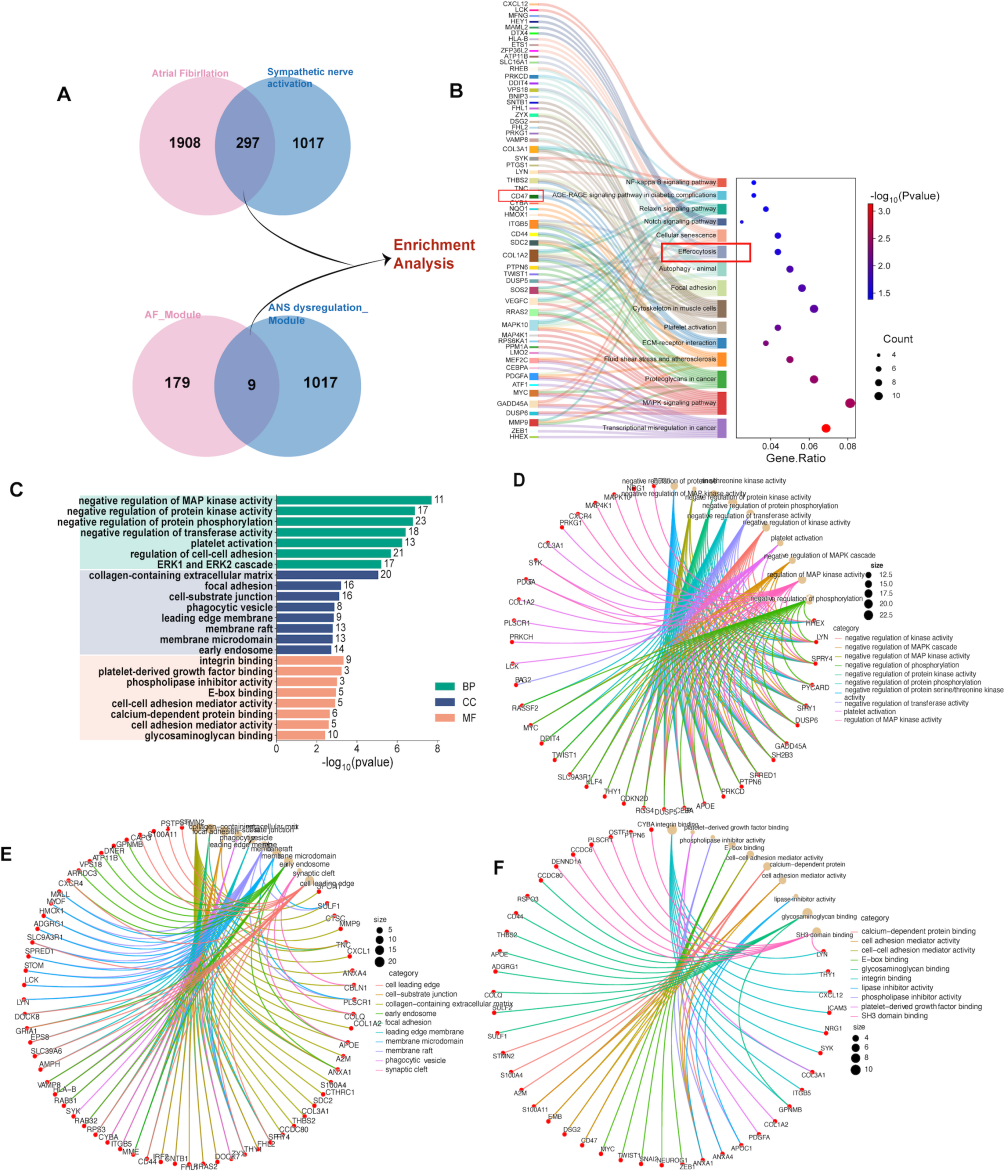
The enrichment analysis of ANS dysregulation-related AF genes. **(A)** A gene set integrating shared DEGs and overlapping key modules genes between AF and sympathetic nerve activation. **(B)** KEGG items of ANS dysregulation-related AF gene set. **(C–F)** The overall of GO items of ANS dysregulation-related AF gene set and cnet plot of BP, CC and MF, respectively. AF atrial fibrillation, ANS autonomic nervous system, BP biological process, CC cellular component, GO gene ontology, KEGG Kyoto encyclopedia of genes and genomes, MF molecular function.

Further integrating the shared DEGs previously identified from the AF and sympathetic nerve activation datasets, a preliminary set of 304 common genes associated with ANS dysregulation-related AF was obtained and employed for enrichment analysis (**Figure 4A**; **Supplementary Table 2**). Primarily enriched genes and significant KEGG pathways include mitogen-activated protein kinase (MAPK) signaling pathway, proteoglycan glycosylation, efferocytosis, and cellular autophagy (**Figure 4B**). In the Gene Ontology (GO) analysis of the shared genes, the top 10 most significant terms in each of the three categories are presented in **Figure 4C**. The enrichment networks for biological processes (BP), cellular components (CC), and molecular functions (MF) are illustrated in **Figures 4D–F**. Key BPs included negative regulation of protein serine kinase activity, negative regulation of MAPK cascade, and negative regulation of protein phosphorylation. CCs comprised collagen-containing extracellular matrix (ECM), and phagocytic vesicle. MFs involved integrin binding, calcium-dependent protein binding, and glycosaminoglycan binding.

### 3.3 Identification of shared hub genes based on machine learning algorithms

**Figure 5:**
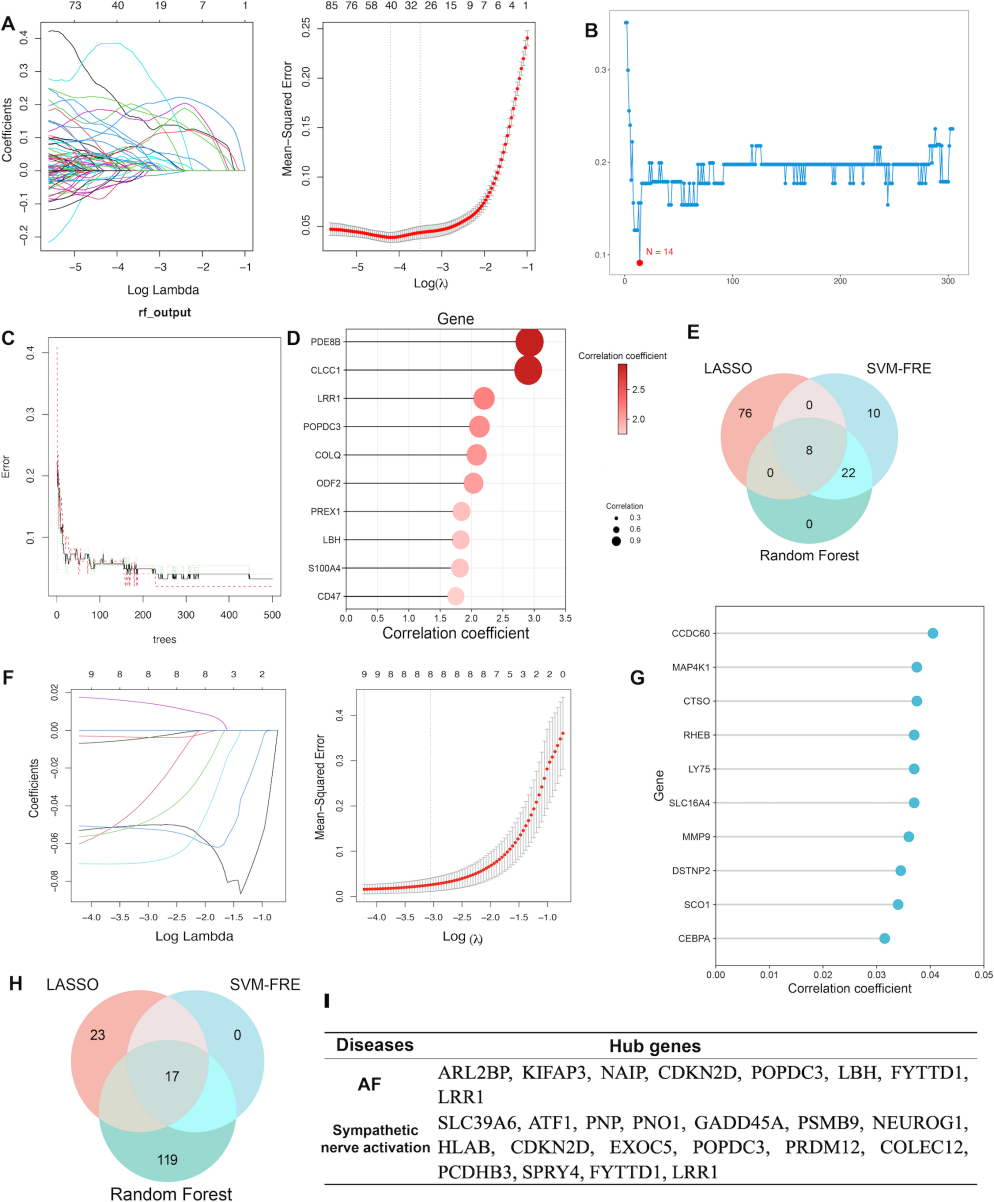
Integrating machine learning algorithms for hub-genes of ANS dysfunction-related AF identification. **(A)** LASSO algorithm analysis for identifying core AF genes. **(B)** SVM-RFE determination of optimal gene subset size for AF. **(C, D)**. Random forest modeling of AF genes (500 decision trees) and display of top 10 genes. **(E)** Intersection of three machine learning algorithms to identify core AF genes. **(F)** LASSO algorithm analysis for identifying core ANS-related genes. **(G)** Random forest modeling of ANS-related genes and display of top 10 genes. **(H)** Intersection of machine learning algorithms to identify core ANS-related genes. **(I)** Hub genes shared between AF and ANS analyzes.

Integrating the gene sets of DEGs and co−expression module genes between AF and sympathetic nerve activation datasets, three machine learning approaches were employed to further identify hub-genes of ANS dysregulation-related AF (**Supplementary Table 3**). In the AF analysis, based on the LASSO coefficient trajectory and optimal tuning parameters, the regularization coefficient minimizing the mean squared error was selected (λ = 0.003690142), yielding 84 genes (**Figure 5A**). When the feature subset size was set to N = 14, the SVM model achieved optimal performance, with 10−fold cross−validation error reaching its minimum (**Figure 5B**). In RF models, the top 30 genes ranked by the Gini index were selected as significant features, and the top 10 genes are displayed (**Figures 5C, D**). The intersection of genes identified by the three models resulted in 8 shared genes for AF (**Figure 5E**). For the ANS analysis, LASSO regression was performed using binary classification for prediction due to the smaller sample size, and SVM analysis was not applicable. Setting λ = 0.01545983 in the LASSO algorithm identified 17 feature genes (**Figure 5F**). RF modeling results showed that with a Gini index significance threshold of 0.02, 40 genes were obtained, and the top 10 are presented (**Figure 5G**). The intersection of genes from the two models yielded 17 core ANS feature genes (**Figure 5H**).

Finally, cyclin−dependent kinase inhibitor 2D (CDKN2D), popeye domain−containing protein 3 (POPDC3), forty−two−3hree domain−containing 1 (FYTTD1), and leucine−rich repeat−containing protein 1 (LRR1) common to both AF and ANS analyzes were identified as hub-genes of ANS dysregulation-related AF (**Figure 5I**).

### 3.4 Modeling and evaluating the diagnostic performance for AF

**Figure 6:**
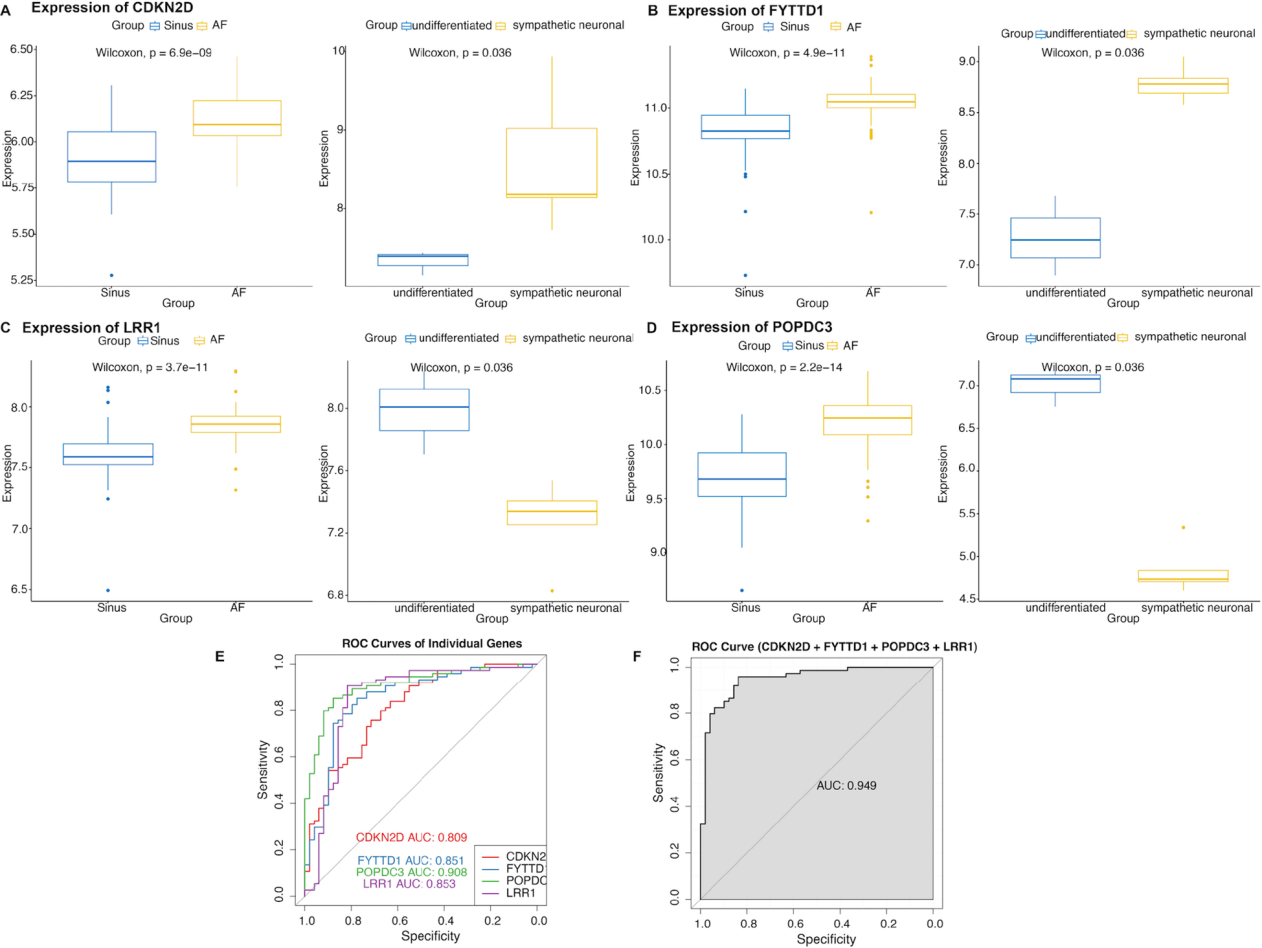
The diagnostic efficacy of hub-genes of ANS dysfunction-related AF. **(A–D)** Comparison of CDKN2D, FYTTD1, LRR1, POPDC3 transcriptional levels between groups in in AF and cardiac sympathetic activation datasets. **(E–F)** ROC curves of CDKN2D, FYTTD1, LRR1, and POPDC3 as single-gene and integrated diagnostic markers for AF.

As compared in **Figures 6A–D**, the transcriptional levels of CDKN2D, FYTTD1, LRR1, and POPDC3 were upregulated in the AF group. In the ANS group, CDKN2D and FYTTD1 exhibited the same upregulation trend, whereas LRR1 and POPDC3 showed downregulated transcription following sympathetic nerve activation (*P* < 0.05). Then, individual ROC curves were plotted for each gene to evaluate the diagnostic efficacy of these four hub-genes for AF (**Figure 6E**). It is indicated that POPDC3 performed the best (AUC = 0.908), while the AUC values of the remaining genes also exceeded 0.80, demonstrating good predictive ability for AF diagnosis. Furthermore, a regression model constructed based on the hub-genes achieved an AUC of 0.949 (**Figure 6F**), which was superior to single-gene predictions, thereby improving diagnostic performance.

### 3.5 Single gene set enrichment analysis and immune infiltration analysis

**Figure 7:**
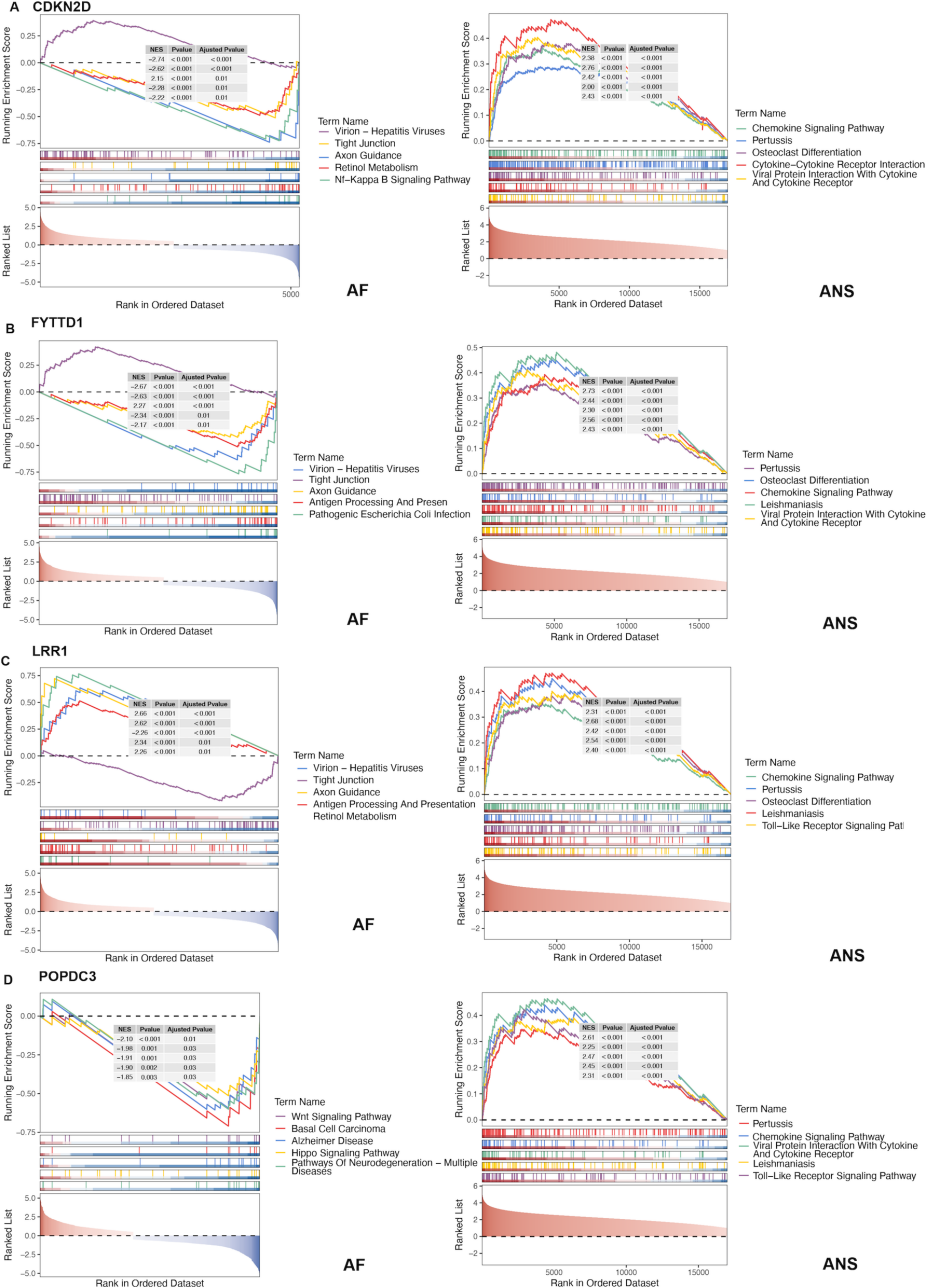
Single GSEA analysis of hub genes for ANS dysfunction-related AF. **(A–D)**. GSEA results of CDKN2D, FYTTD1, LRR1, and POPDC3 in AF and sympathetic activation datasets.

The GSEA was performed on the four hub genes respectively. Significantly enriched pathways mainly included tight junction in epithelial cells, axon guidance, chemokine signaling pathway, toll-like receptor (TLR) pathway, retinol metabolism, nuclear factor-kappa B (NF-κB) signaling pathway, Hippo signaling pathway, Wnt signaling pathway (**Figure 7**). Notably, the marked enrichment of the chemokine and TLR signaling pathways suggests a potential amplification effect driven by immune-mediated autonomic and atrial remodeling.

**Figure 8:**
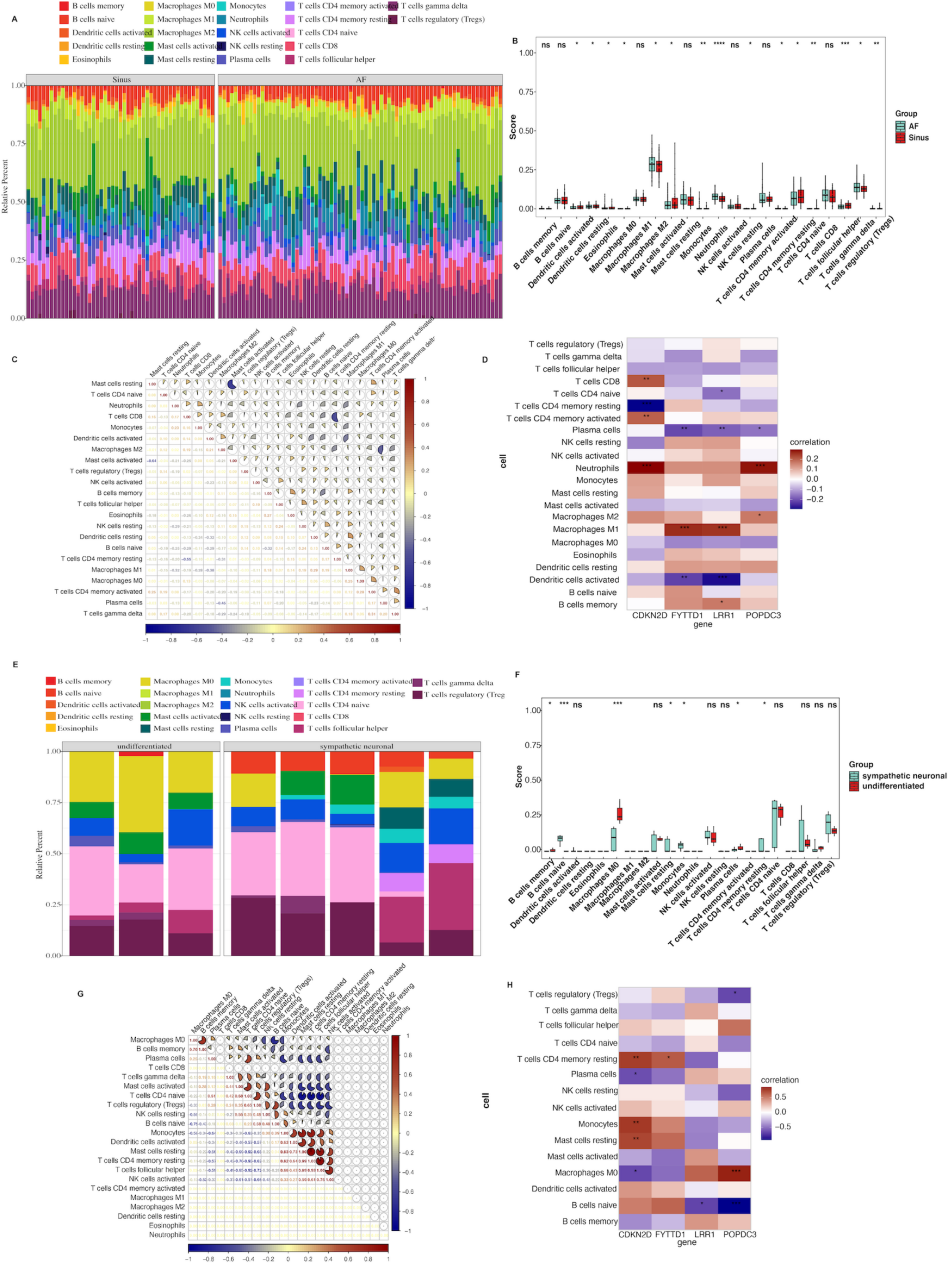
Immune infiltration analysis of AF and ANS datasets. **(A)** Relative proportions of immune cell types among AF cohorts. **(B)** Box plot comparing intergroup differences in immune infiltration in the AF cohort. **(C)** Correlation analysis among immune cell types in AF samples. **(D)** Heatmap of Spearman correlation analysis between hub genes and immune cell types in AF samples. **(E)** Relative proportions of immune cell types among ANS samples. **(F)** Comparison of intergroup differences in immune infiltration in the ANS samples. **(G)** Correlation analysis among immune cell types in ANS samples. **(H)** Heatmap of correlation analysis between hub genes and immune cell types in ANS samples. Compared with the control group, *P < 0.05 and **P < 0.01.

Further immune infiltration revealed that both AF and ANS groups exhibited a reduction in M0 macrophages alongside enhanced macrophage polarization, mast cell activity, and neutrophil infiltration (**Figures 8A, E**). Box plots quantified intergroup changes, showing an increase in both M1 pro-inflammatory macrophages and M2 repair macrophages, as well as activated naive CD4^+^ T cells and CD4 memory T cells (**Figures 8B, F**). Subgroup correlations in AF cohorts indicated strong positive relevance between γδ T cells and CD4^+^ T cells, and negative correlations between CD4 memory resting T cells and CD8^+^ T-cell activity (**Figure 8C**). In ANS samples, dendritic cell activation positively correlated with M1 polarization while naive B cells inversely correlated with M0 macrophages (**Figure 8G**). Correlation analysis revealed pro-inflammatory roles for CDKN2D and FYTTD1 (associated with M1 macrophages/neutrophils) in AF, whereas LRR1 correlated with M2 macrophages and CD4 memory T cells (**Figure 8D**). In ANS samples, CDKN2D and LRR1 were linked with unpolarized macrophage differentiation, CDKN2D and FYTTD1 inversely correlated with resting CD4 memory T cells, and POPDC3 negatively correlated with regulatory T cells (**Figure 8H**).

### 3.6 Molecular docking between valsartan and hub genes

**Figure 9:**
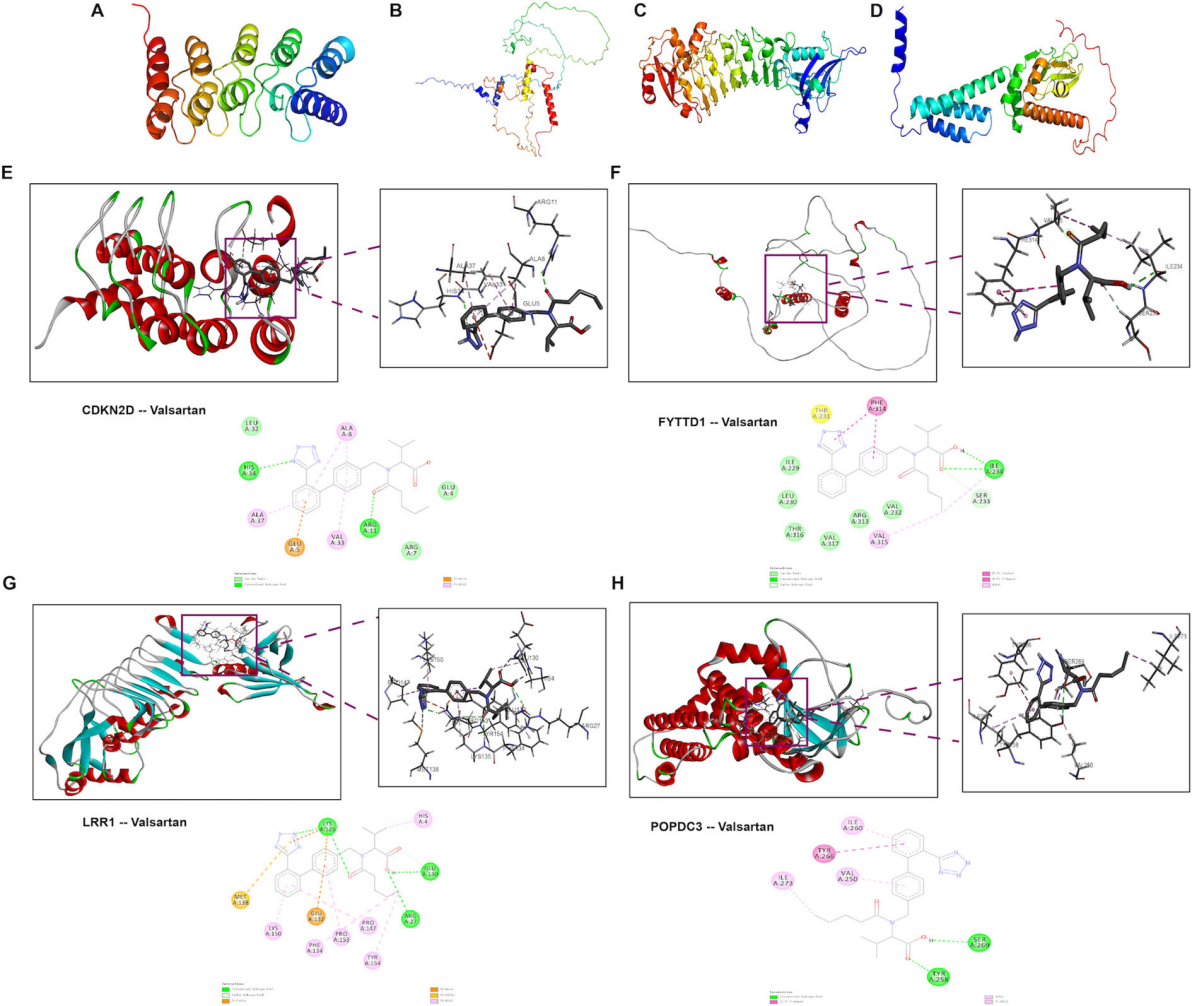
Molecular docking between valsartan and targets of ANS dysregulation-related AF. **(A–D)** Structures of CDKN2D, FYTTD1, LRR1, and POPDC3 predicted by AlphaFold, with colors indicating the predicted confidence levels (from red for very low confidence, through yellow and light blue, to dark blue for very high confidence). **(E–H)** Visualization of docking results for valsartan with CDKN2D, FYTTD1, LRR1, and POPDC3, presented using PyMOL and discovery studio.

Given the importance of maintaining optimal blood pressure for AF prevention, the 2024 ESC guidelines for the management of AF recommend angiotensin-converting enzyme inhibitors (ACEIs) or angiotensin receptor blockers (ARBs) as a first-line therapy (3). Compared to other antihypertensive drugs, valsartan has been proven a more distinct reduction in susceptibility to AF and mitigate atrial remodeling (36, 37). Separate clinical studies have also demonstrated that valsartan significantly improves cardiac ANS function by increasing parasympathetic tone (38). To preliminarily investigate the mechanism by which valsartan prevents AF, valsartan was docked with each hub genes for ANS dysfunction-related AF in this study (**Figure 9**). Predicting the structures of the four potential targets, the calculated binding energies between valsartan and CDKN2D, FYTTD1, LRR1, and POPDC3 were -6.4 kcal/mol, -5.9 kcal/mol, -6.8 kcal/mol, and -6.3 kcal/mol, respectively, indicating favorable binding affinity (39).

### 3.7 Valsartan mitigates the autonomic remodeling in mice with Ang II-induced AF

**Figure 10:**
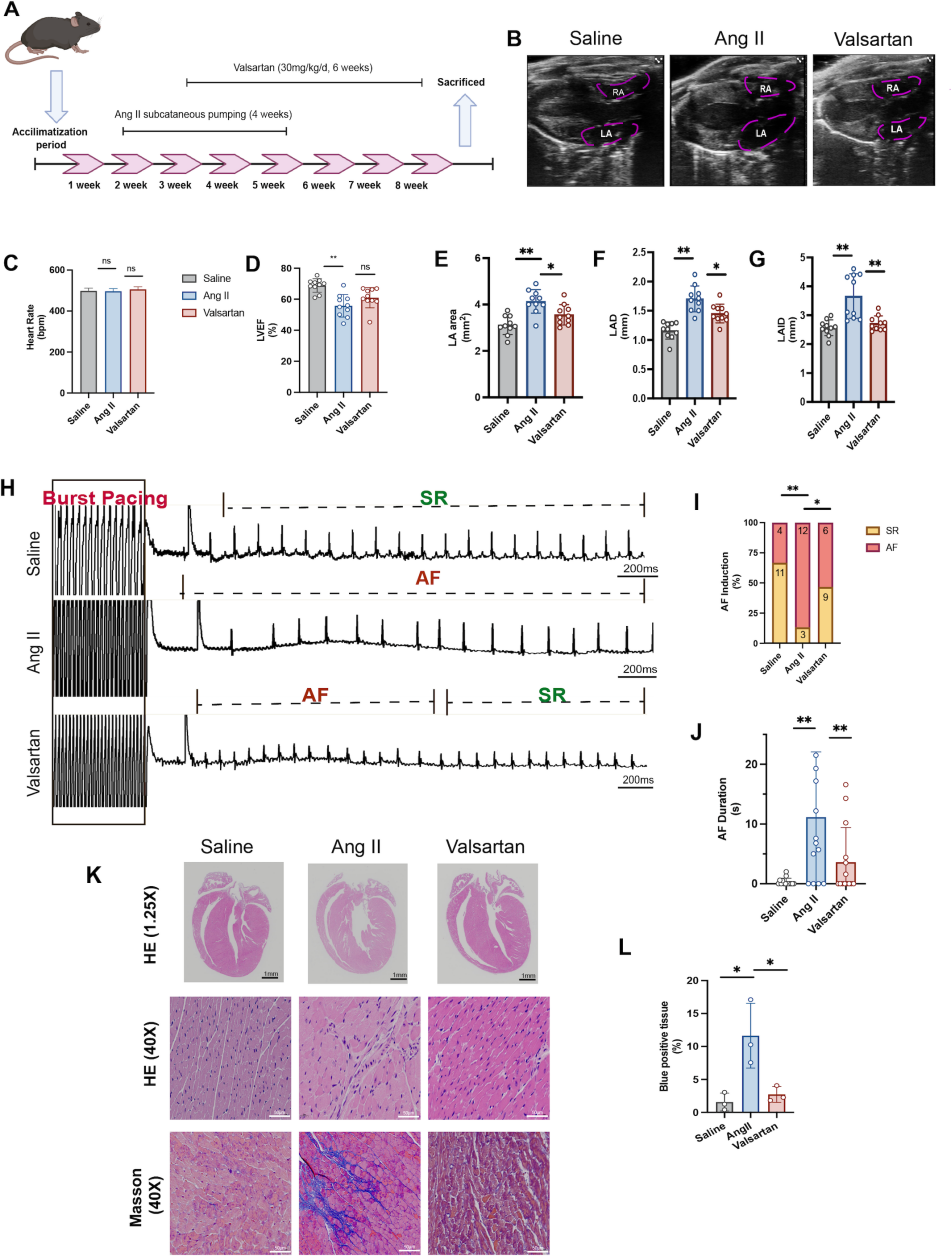
Valsartan attenuates Ang II–induced left atrial remodeling and reduces AF susceptibility in mice. **(A)** Study design. **(B)** Representative transthoracic echocardiography images showing the right atrium (RA) and left atrium (LA) in saline, Ang II, and valsartan groups. **(C–G)** Echocardiographic measurements (n = 10): **(C)** heart rate (HR), **(D)** left ventricular ejection fraction (LVEF), **(E)** LA area, **(F)** LA diameter (LAD), and **(G)** LA internal diameter (LAID). Each dot represents a mouse, bars indicate mean ± SD. **(H)** The representative electrocardiograms during burst pacing–based AF induction. **(I)** The proportion of AF inducibility and **(J)** AF duration (n = 15). **(K)** Representative histology of HE (1.25× and 40×) and Masson (40×) staining (n = 6). **(L)** Quantification of cardiac fibrosis as blue-positive area. *P < 0.05 and **P < 0.01 compared with the Ang II group.

After acclimatization, mice received Ang II infusion via subcutaneous minipump and valsartan administration by oral gavage (**Figure 10A**). Consistent with atrial remodeling, echocardiography demonstrated significant enlargement of the left atrium (LA) in Ang II–treated mice (**Figure 10B**). While heart rate was comparable among groups (**Figure 10C**), Ang II reduced left ventricular systolic function as indicated by the lower LVEF (**Figure 10D**). Ang II also markedly increased LA size, including LA area, LAD and LAID, and valsartan treatment partially mitigated the atrial dilation (**Figures 10E–G**). Then, AF was induced by administering burst pacing among groups (**Figure 10H**). Ang II markedly increased AF susceptibility, as evidenced by higher AF inducibility and prolonged AF duration (**Figures 10I, J**). Importantly, valsartan significantly reduced both AF inducibility and duration compared with Ang II, indicating an attenuation of the arrhythmogenic substrate (**Figures 10I, J**). Histological assessment further supported structural remodeling. Ang II induced prominent cell disarray, inflammatory infiltration, and myocardial fibrosis, whereas valsartan significantly alleviated these above pathological alterations (**Figures 10K, L**).

**Figure 11:**
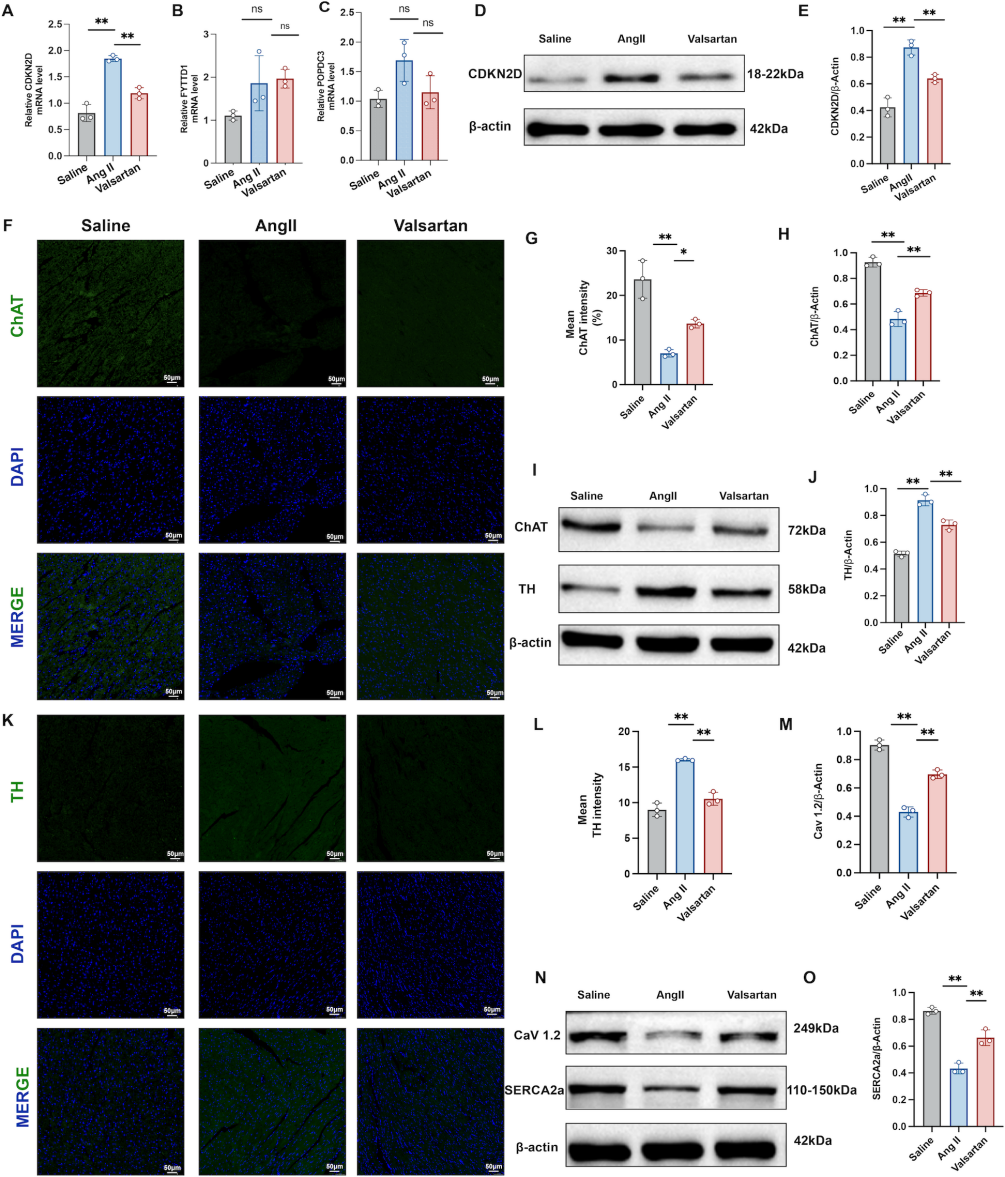
Valsartan attenuates Ang II-induced autonomic remodeling in mice with AF. **(A–C)** RT-qPCR analysis of hub-genes of ANS dysregulation-related AF (CDKN2D, FYTTD1, and POPDC3) mRNA levels in atrial tissues. **(D, E)** Immunoblot images and quantification of CDKN2D protein levels. **(F, G)** Representative immunofluorescence images and quantification of fluorescence intensity of atrial sections stained for ChAT (green) with DAPI (blue) (n=3).Scale bar, 50 μm. **(H–J)** Immunoblot images and quantification of ChAT and TH protein levels. **(K, L)** Representative immunofluorescence images and quantification of fluorescence intensity of atrial sections stained for TH (green) with DAPI (blue) (n=3). **(M–O)** immunoblot images and quantification of Ca^2+^ transmission protein levels (CAV1.2 and SERCA2a). *P < 0.05 and **P < 0.01 compared with the Ang II group.

The transcription levels of four ANS-dysregulation-related AF hub genes were quantified by the RT-qPCR (**Table 1**). Notably, LRR1 expression was below the detection threshold in atrial samples and could not be reliably analyzed. As predicted in silico, Ang II increased the CDKN2D, FYTTD1, and POPDC3 at mRNA levels, and valsartan administration partially attenuated this induction. The trends in FYTTD1 and POPDC3 did not reach statistical significance (**Figures 11A–C**). Ang II also robustly increased CDKN2D protein levels, which were normalized by valsartan (**Figure 11D**).

In parallel, Ang II provoked a clear autonomic remodeling phenotype in the atrial. Immunofluorescence and immunoblotting demonstrated a loss of the parasympathetic marker choline acetyltransferase (ChAT) following Ang II, which was partially restored by valsartan (**Figures 11F–I**). Conversely, Ang II enhanced the sympathetic activity, evidenced by increased tyrosine hydroxylase (TH) immunofluorescence and protein abundance, this increase was significantly blunted with valsartan treatment (**Figures 11I–L**). Simultaneously, Ang II was accompanied by suppression of key Ca²^+^ handling components, including L-type Ca²^+^ channel (CaV1.2) and sarcoplasmic/endoplasmic Reticulum Ca²^+^-ATPase 2a (SERCA2a), and valsartan substantially rescued their expression (**Figures 11M–O**), supporting a protective effect against Ang II–driven Ca²^+^-mediated electrical remodeling in the atrium.

### 3.8 Efferocytosis‐mediated inflammation amplifies ANS dysregulation–related atrial remodeling

**Figure 12:**
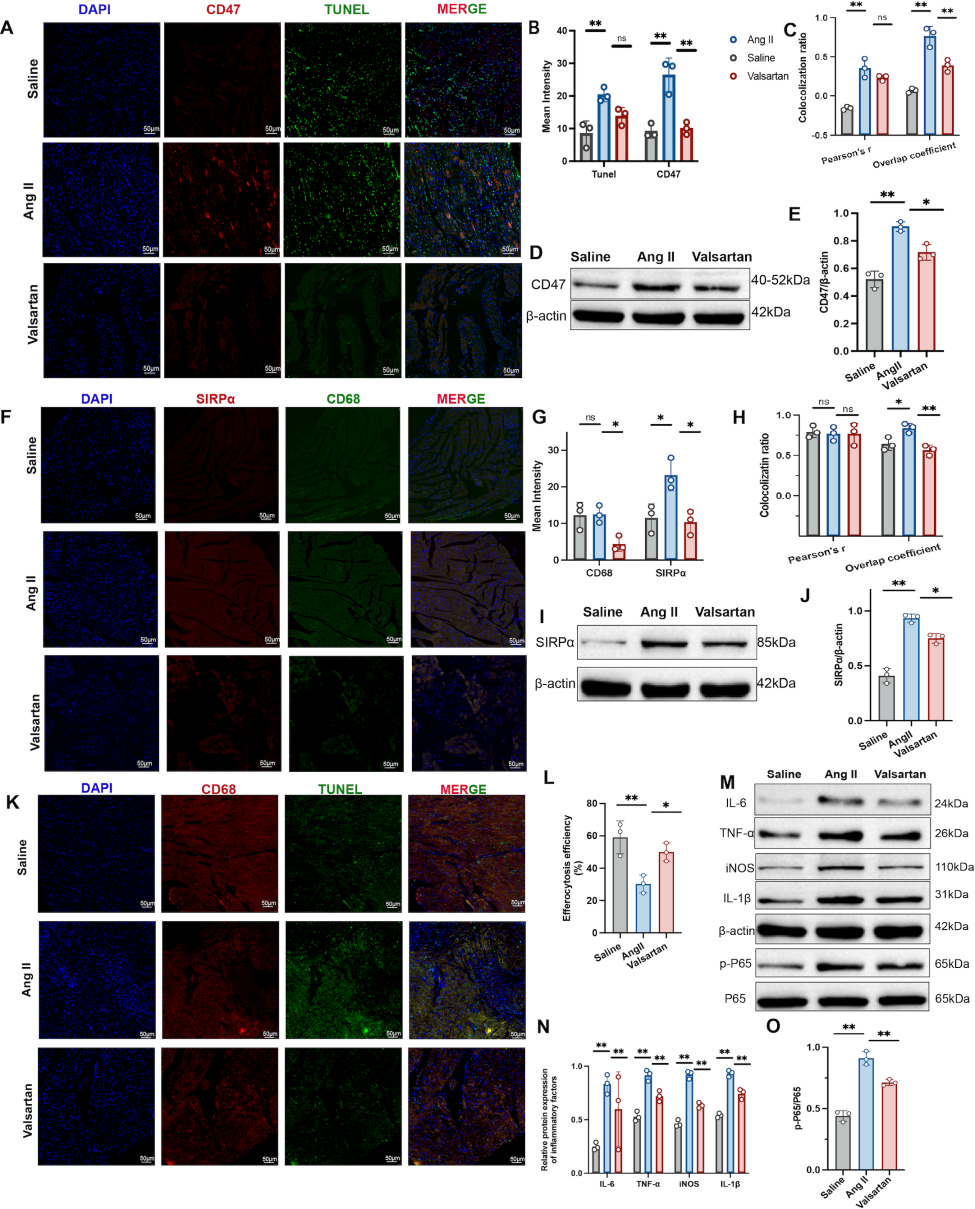
CD47–SIRPα mediated efferocytosis deficiency drives pro-inflammation in Ang II–induced AF. **(A)** Representative immunofluorescence images of atrial sections stained for nuclei (DAPI, blue), CD47 (red), and apoptotic cells (TUNEL, green) in Saline, Ang II, and valsartan groups. Scale bar, 50 μm. **(B, C)** Quantification of mean fluorescence intensity and colocalization for TUNEL and CD47. **(D, E)** Immunoblot images and quantification of CD47 protein levels. **(F)** Representative immunofluorescence images of atrial sections stained for nuclei (DAPI, blue), SIRPα (red), and macrophages (CD68, green) in the three groups. Scale bar, 50 μm. **(G, H)** Quantification of mean fluorescence intensity and colocalization for CD68 and SIRPα. **(I, J)** Immunoblot images and quantification of SIRPα protein levels. **(K)** Representative immunofluorescence images depicting CD68 (red) and TUNEL (green) to assess macrophage-associated apoptotic signals in atrial sections; nuclei are counterstained with DAPI (blue). Scale bar, 50 μm. **(L)** Quantification of efferocytosis efficiency, estimated as the percentage of CD68-associated TUNEL-positive signals among total TUNEL-positive signals in the analyzed atrial fields. **(M–O)** Immunoblot images and quantification of inflammatory responses factors (IL-6, TNF-α, IL-1β, iNOS) and p-P65. *P < 0.05 and **P < 0.01 compared with the Ang II group.

Indicated by the bioinformatics analysis, including WGCNA, function enrichment, and immune infiltration analyzes, the CD47-mediated efferocytosis pathway was identified as a putative mechanism linking ANS dysregulation to atrial remodeling in AF. Histological examination of atrial sections from Ang II-infused mice revealed a marked increase in TUNEL-positive apoptosis together with induced CD47 immunoreactivity (**Figures 12A, B**). Moreover, Ang II significantly enhanced the spatial association between CD47 and TUNEL signals, as reflected by increased colocalization metrics (**Figures 12A, C**), and this was corroborated by immunoblotting showing elevated CD47 protein abundance (**Figures 12D, E**). Treatment with valsartan effectively blunted these effects, suppressing both apoptosis and CD47 upregulation while reducing CD47–TUNEL overlap (**Figures 12A–E**).

Parallelly, Ang II augmented the “don’t-eat-me” receptor axis in the phagocyte compartment. Immunofluorescence demonstrated elevated signal regulatory protein α (SIRPα) and altered CD68 signal intensity, with strengthened SIRPα–CD68 spatial coupling under Ang II exposure (**Figures 12F–H**). Immunoblotting further confirmed a significant increase in SIRPα protein, which was partially normalized by valsartan (**Figures 12I, J**). rom a functional standpoint, Ang II severely compromised efferocytosis efficiency despite a higher apoptotic burden, pointing to a profound defect in the clearance of dying cells (**Figures 12K, M**). This defect was accompanied by heightened inflammatory activation, evidenced by elevated cytokines including IL-6, TNF-α, and IL-1β, M1 macrophage polarization, and enhanced NF-κB signaling (**Figures 12L–O**). Crucially, valsartan partially restored efferocytosis efficiency and the downstream inflammatory mediator expression and NF-κB activation (**Figures 12M–O**).

Collectively, these data suggest that CD47–SIRPα-dependent efferocytosis failure and subsequent pro-inflammatory amplification are highly associated with ANS dysregulation in the Ang II model. This pathway represents a potential downstream effector module exacerbating atrial electrophysiological and structural remodeling.

## 4 Discussion

**Figure 13:**
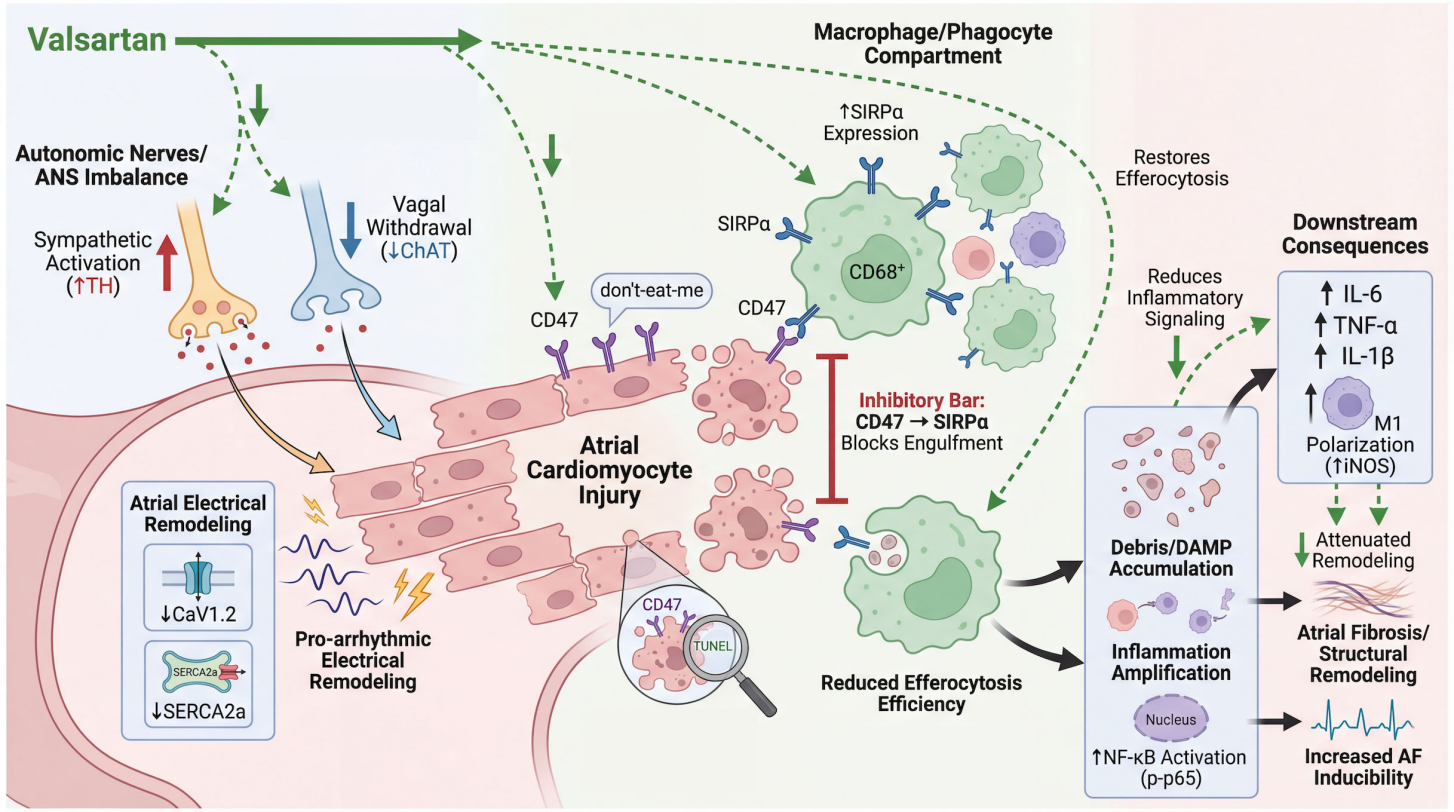
Proposed mechanism of impairing CD47-SIRPα-mediated efferocytosis recognition in AF. Ang II induces ANS imbalance with Ca²^+^-handling remodeling, promoting atrial cardiomyocyte injury, CD47 and macrophage SIRPα upregulation. CD47–SIRPα “don’t-eat-me” signaling inhibits engulfment, reducing efferocytosis efficiency and driving debris accumulation.

Sympathetic and vagal nerves constitute a dense network in the atria and dynamically modulate ion channel activity through the release of norepinephrine and acetylcholine, facilitating depolarization and shortening action potential duration (APD) (14, 40). But it does not fully account for why ANS imbalance often coexists with persistent atrial inflammation and fibrotic remodeling. The present study extends the autonomic framework by proposing that ANS dysregulation is an electrical trigger, as well as an upstream amplifier of inflammatory loops, and that failure of efferocytosis, centering on the CD47–SIRPα “don’t-eat-me” axis, represents a plausible mediator linking autonomic dysfunction to sustained atrial injury (**Figure 13**). This concept addresses a clinically relevant gap, since AF pathogenesis is widely recognized to involve autonomic imbalance while there are yet no clinically authorized antiarrhythmic drugs specifically targeting ANS modulation.

First, clinical evidence supporting an autonomic phenotype was provided by targeted metabolomics profiling of 39 neurotransmitters. It revealed the pattern of elevated monoaminergic and reduced cholinergic neurotransmitters, implying sympathetic excitation with relative vagal suppression in AF. Although circulating neurotransmitters are systemic proxies rather than direct measures of intracardiac neural activity, this signature supports an ANS-dysfunction–related AF phenotype. Moreover, AF cohorts also showed higher biomarker evidence of chronic low-grade inflammation. To delineate the molecular interaction, bioinformatics and machine learning approaches were subsequently applied. 304 common genes associated with ANS-dysfunction-related AF were defined by integrating DEGs and shared WGCNA co-expression modules from AF atrial tissue and sympathetic neuron differentiation. Both pathway enrichment and WGCNA modules consistently identified CD47 as a prominent shared gene between AF and sympathetic activation. Notably, CD47-mediated efferocytosis was one of the significantly enriched terms (**Figure 4B**) (41). Immune infiltration analysis further indicated reduced M0 macrophages with enhanced macrophage polarization, mast cell and neutrophil activity. In this hypothesis, impaired efferocytosis might explain how autonomic stress driving atrial remodeling through failure of tissue homeostasis depending on recognition and clearance of apoptotic cells to avert secondary necrosis, damage-associated molecular patterns (DAMP) accumulation, and persistent inflammation (42). Collectively, we propose a specific cell crosstalk framework: increased CD47 on apoptotic cardiomyocytes can bind to its ligand SIRPα on infiltrating macrophages, imposing an inhibitory checkpoint on efferocytosis and thereby promoting debris accumulation and sustained inflammatory activation (43–45).

The *in vivo* Ang II model supported this logic. Ang II infusion was accompanied by impaired cardiac function, significant atrial injury and fibrosis, and an increased susceptibility to burst pacing–induced atrial arrhythmia. At the molecular level, TH, a canonical sympathetic nerve marker, was upregulated, whereas a marker of parasympathetic innervation, ChAT was reduced, indicating Ang II–driven atrial autonomic remodeling. In parallel, key Ca²^+^-handling proteins were suppressed, including L-type Ca²^+^ channel protein and SERCA2a, consistent with impaired Ca²^+^ influx and Ca²^+^ reuptake that would be expected to destabilize atrial excitability (35). Atrial sections from Ang II–infused mice showed increased TUNEL-positive apoptosis together with induction of CD47 and enhanced spatial association between CD47 and TUNEL signals. In the phagocyte compartment, Ang II augmented the don’t-eat-me axis, with strengthened SIRPα–CD68 spatial coupling, and a confirmed rise in SIRPα protein. Functionally, Ang II produced a pronounced impairment in efferocytosis efficiency despite the increased apoptotic load, indicating defective clearance rather than insufficient availability of targets for phagocytosis. This efferocytosis defect coincided with heightened inflammatory activation, including increased M1 macrophage polarization and enhanced NF-κB signaling. Taken together, these results suggest a potential pathophysiological link in which autonomic stress elevates apoptotic burden while CD47–SIRPα signaling simultaneously raises for macrophage engulfment deficiency, converting tissue injury into a self-sustaining inflammatory state that can further destabilize atrial electrophysiology and structure.

Valsartan emerged in this study as a clinically relevant perturbation of the proposed axis. 2024 ESC guidelines supports the use of ACEIs/ARBs as first-line therapy in AF management for blood pressure optimization, and valsartan has been reported to reduce AF susceptibility and mitigate atrial remodeling, with preclinical studies suggesting improved cardiac ANS function via increased parasympathetic tone (3, 36, 46–48). *In silico* molecular docking further indicated favorable binding affinities between valsartan and the hub-gene targets (binding energies approximately −5.9 to −6.8 kcal/mol), which can be framed as hypothesis-generating support for potential molecular interactions. In the Ang II model, valsartan suppressed TUNEL signal and CD47 upregulation, reduced CD47–TUNEL overlap, partially normalized SIRPα levels, and restored efferocytosis efficiency, accompanied by dampened inflammatory mediator expression and reduced NF-κB activation. These findings suggest that valsartan may act beyond blood pressure control by mitigating the broader Ang II–ANS–immune remodeling network. Nevertheless, the valsartan-induced improvement in efferocytosis should be interpreted cautiously. Because valsartan broadly suppresses apoptosis, oxidative and inflammatory stress, macrophage activation, and tissue fibrosis, the reduced CD47, SIRPα expression and increased CD68-associated TUNEL clearance may partly reflect a secondary consequence of a less injurious atrial microenvironment rather than a direct CD47–SIRPα-specific effect (49, 50).

Admittedly, several limitations in the current research should be noted. First, the human neurotransmitter profiling was performed on fasting plasma from an observational cohort, which supports association with an autonomic imbalance but does not establish causality or exclude confounding by comorbidity burden or systemic inflammation. Secondly, the relatively limited samples, particularly within the ANS dataset, inherently introduces the risk of model overfitting. The discrepancy between in silico predictions and *in vivo* results underscores that these hub genes should be viewed strictly as preliminary candidates requiring further stringent validation in larger, independent cohorts. Thirdly, the Ang II infusion model provides experimental leverage but represents a specific remodeling context and does not exhaust the etiological diversity of ANS-dysregulation-related AF. Finally, the current study primarily relies on multi-omics study and *in vivo* observation. Specifically, the lack of loss-of-function or gain-of-function experiments inherently the direct mechanistic necessity of the CD47-SIRPα axis remains to be fully elucidated. Future longitudinal studies and targeted mechanistic investigations using transgenic models or specific inhibitors are warranted to definitively validate the causal pathways underlying this proposed axis.

## 5 Conclusion

In conclusion, a multi-omics study integrating targeted neurotransmitter metabolomics, transcriptomics, together with *in vivo* validation identified a novel ANS–immune–atrial remodeling axis that is highly associated with increased AF susceptibility. Shared hub genes (CDKN2D, LRR1, FYTTD1, and POPDC3) and a CD47-centered efferocytosis emerged as key links between autonomic imbalance and sustained atrial injury and pro-arrhythmic substrate formation. In the Ang II model, autonomic remodeling and Ca²^+^-handling impairment, increased apoptotic burden, and activation of the CD47–SIRPα “don’t-eat-me” axis coincided with defective efferocytosis and heightened inflammatory signaling. These observations suggest a potential crosstalk from neural imbalance to immune dysregulation and atrial remodeling. Importantly, valsartan attenuated atrial remodeling and AF inducibility while partially normalizing autonomic markers and restoring efferocytosis efficiency with reduced downstream inflammation. Together, these findings highlight the CD47-SIRPα pathway as a promising candidate for immune-targeted strategies in AF management.

## Data Availability

The original contributions presented in the study are included in the article/**Supplementary Material**. Further inquiries can be directed to the corresponding authors.
