# Supplementary material for: Defective efferocytosis links autonomic dysfunction and atrial fibrillation: multi-omics integration and in vivo validation
Source: Front Immunol. 2026 May 22;17:1818859. doi: 10.3389/fimmu.2026.1818859 (PMC13237436; doi:10.3389/fimmu.2026.1818859)
Supplement: Supplementary file 1 [file Table1.docx]

**Supplementary Table S1. Demographics of AF and SR participants**

| **Characteristics** | | **SR (n = 30)** | | **AF (n = 70)** | ***X^2^/Z/t*** | ***P*** |
| --- | --- | --- | --- | --- | --- | --- |
| Gender (male/female) | | 16/14 | | 38/32 | 0.008 | 0.93 |
| Age, y | | 54.63 ± 11.37 | | 57.10 ± 10.12 | -1.076 | 0.310 |
| Body mass index, kg/m^2^ | | 23.54 ± 2.04 | | 23.44 ± 2.01 | -0.034 | 0.761 |
| Systolic BP, mmHg | | 139.03 ± 18.97 | | 141.89 ± 12.70 | -0.743 | 0.462 |
| Diastolic BP, mmHg | | 72.80 ± 8.97 | | 71.66 ± 10.40 | 0.519 | 0.605 |
| Comorbidities, % | |  | |  |  |  |
| Hypertension | | 14 (46.67) | | 34 (48.57) | 0.031 | 0.861 |
| Hyperlipidaemia | | 19 (63.33) | | 40 (57.14) | 0.333 | 0.564 |
| Type 2 diabetes | | 12 (40.00) | | 18 (25.71) | 2.041 | 0.153 |
| Obstructive sleep apnea | | 11 (36.67) | | 20 (28.57) | 0.643 | 0.422 |
| Heart failure | | 2 (6.67) | | 4 (5.71) | 0.034 | 0.851 |
| Anticoagulants, % | | 8 (26.67) | | 14 (20.00) | 0.544 | 0.461 |
| Antiarrhythmic drugs, % | | 7 (23.33) | | 20 (28.57) | 0.292 | 0.589 |
| β-blockers, % | | 18 (60.00) | | 36 (51.43) | 0.621 | 0.431 |
| ACEIs/ARBs, % | | 12 (40.00) | | 27 (38.57) | 0.018 | 0.893 |
| Non-dihydropyridine CCB, % | | 4 (13.33) | | 11 (15.71) | 0.093 | 0.760 |
| Antidepressants, % | | 3 (10.00) | | 9 (12.86) | 0.162 | 0.687 |
| GLU, mmol/L | 5.70 (4.86, 7.03) | | 5.75 (4.98, 7.66) | | -0.128 | 0.901 |
| hs-CRP, mg/L | 0.76 (0.48, 1.28) | | 1.14 (0.55, 2.11) | | -1.659 | 0.098 |
| HCY, μmol/L | 12.20 (11.70-14.70) | | 14.70 (12.10-16.30) | | -2.347 | 0.019 |
| UA, μmol/L | 365.33 ± 70.01 | | 384.19 ± 96.26 | | -1.083 | 0.283 |
| CHO, mmol/L | 4.35 (3.29, 4.76) | | 3.47 (2.99, 4.11) | | -1.820 | 0.069 |
| TG, mmol/L | 1.32 (1.14, 1.95) | | 1.24 (0.88, 1.57) | | -1.779 | 0.075 |
| HDL-C, mmol/L | 1.07 (1, 1.23) | | 0.97 (0.84, 1.10) | | -2.149 | 0.032 |
| LDL-C, mmol/L | 2.07 (1.71, 2.57) | | 2.62 (2.01, 3.07) | | -1.502 | 0.133 |

Measurement data were expressed as mean ± SD, or as median (interquartile range) [M (P25, P75)], and categorical data were presented as n (%). The significance level was set at α = 0.05 (two-sided), with 95% confidence intervals. Continuous variables were analyzed using an independent-samples *T* test when normally distributed; otherwise, the Mann–Whitney U test was applied. Categorical variables were compared using the χ² test. ACEI, angiotensin converting enzyme inhibitors; ARB, angiotensin receptor blocker; BP, blood pressure; CCB, calcium channel blocker; CHO, total cholesterol; GLU, glucose; HCY, homocysteine; HDL-C, high-density lipoprotein cholesterol; hs-CRP, high-sensitivity C-reactive protein; LDL-C, low-density lipoprotein cholesterol; TG, triglycerides; UA, uric acid.
