# Supplementary material for: Defective efferocytosis links autonomic dysfunction and atrial fibrillation: multi-omics integration and in vivo validation
Source: Front Immunol. 2026 May 22;17:1818859. doi: 10.3389/fimmu.2026.1818859 (PMC13237436; doi:10.3389/fimmu.2026.1818859)
Supplement: Supplementary file 4 [file Table4.docx]

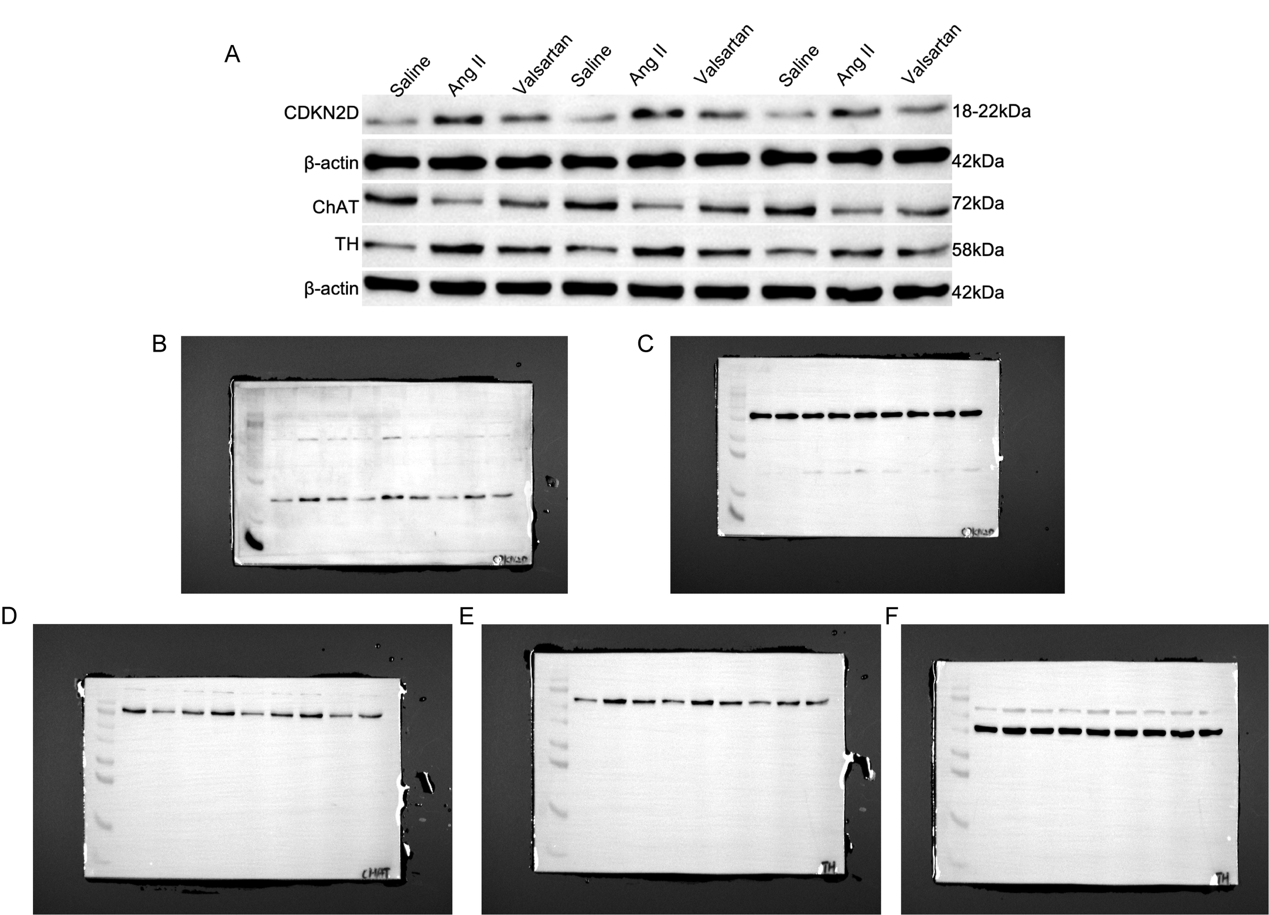


Figure S1. (A) WB strip of CDKN2D, ChAT, and TH with three repeats, respectively. (B-C) The original PVDF membrane of CDKN2D and the relevant β-actin. (D-F) The original PVDF membrane of ChAT, TH and the relevant β-actin.


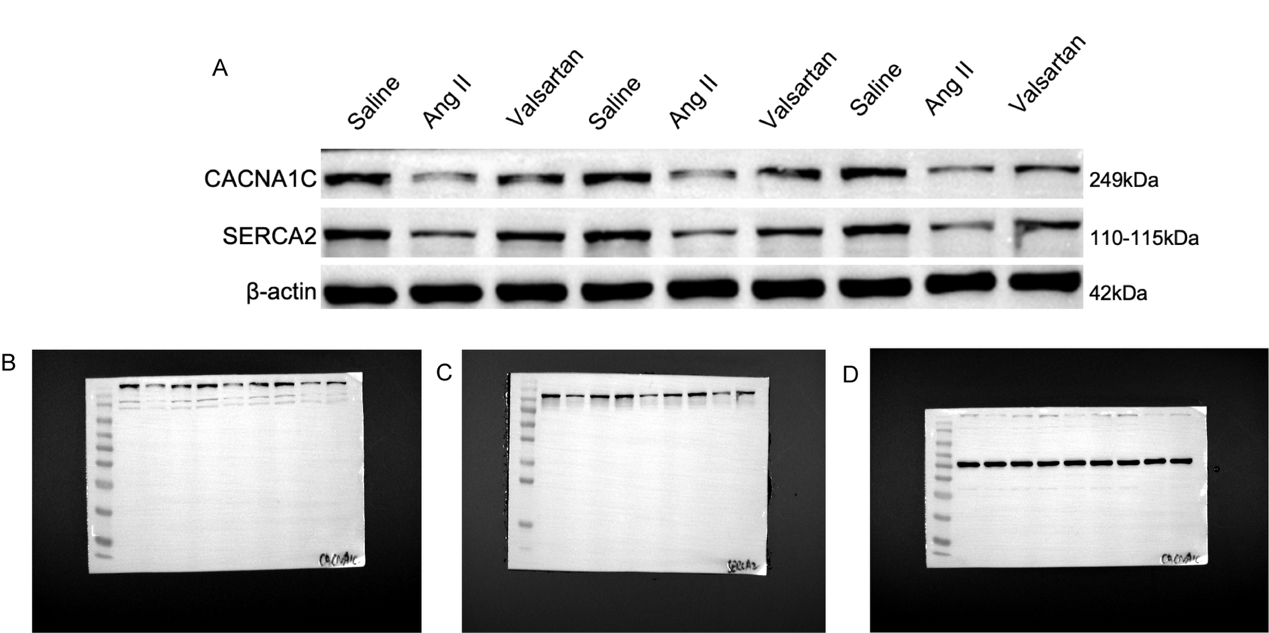


Figure S2. (A) WB strip of CACNA1C and SERCA2 with three repeats, respectively. (B-D) The original PVDF membrane of CACNA1C, SERCA2 and the relevant β actin.


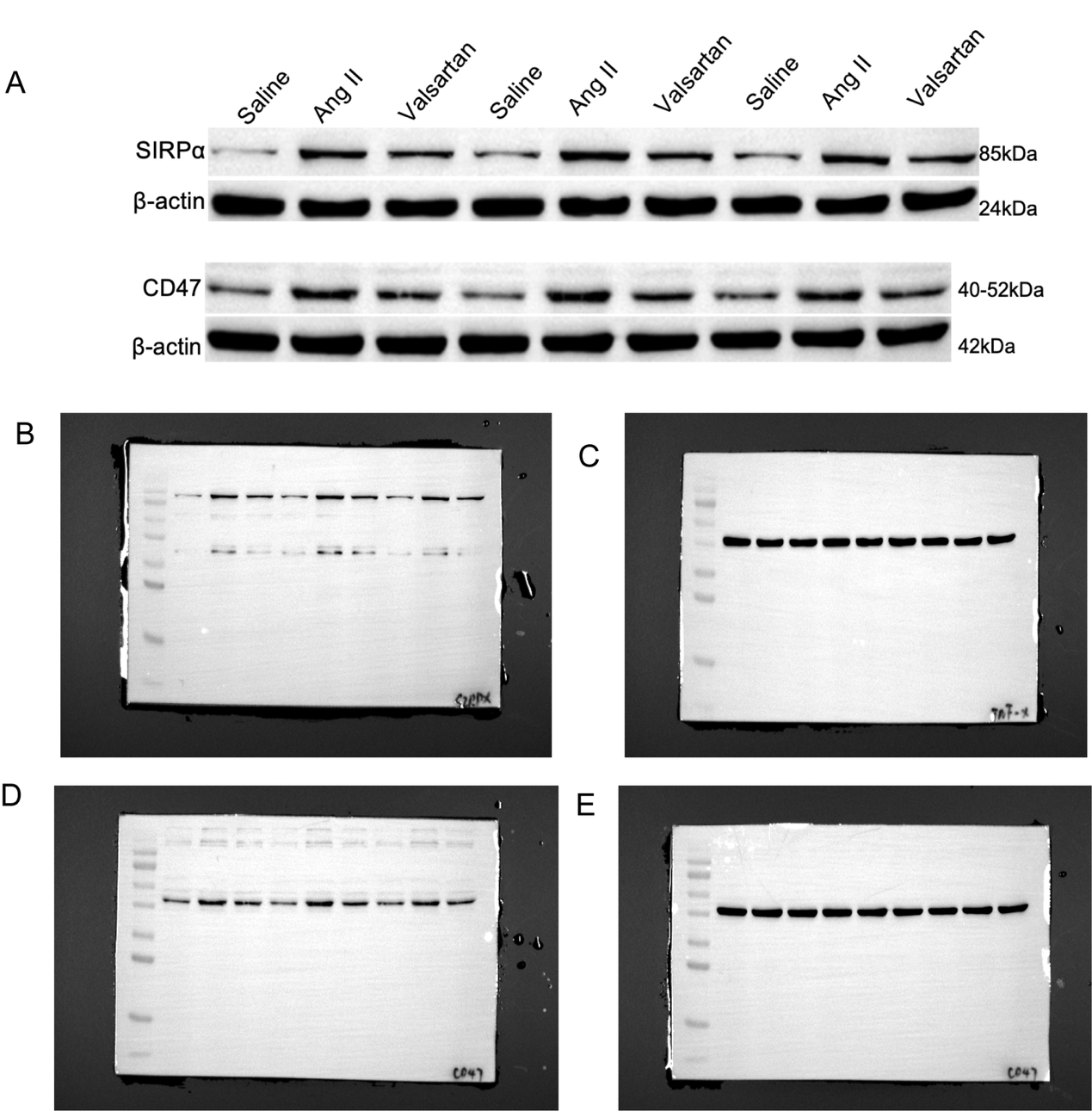


Figure S3. (A) WB strip of SIRPα and CD47 with three repeats, respectively. (B-C) The original PVDF membrane of SIRPα and the relevant β actin. (D-E) The original PVDF membrane of CD47 and the relevant β actin.


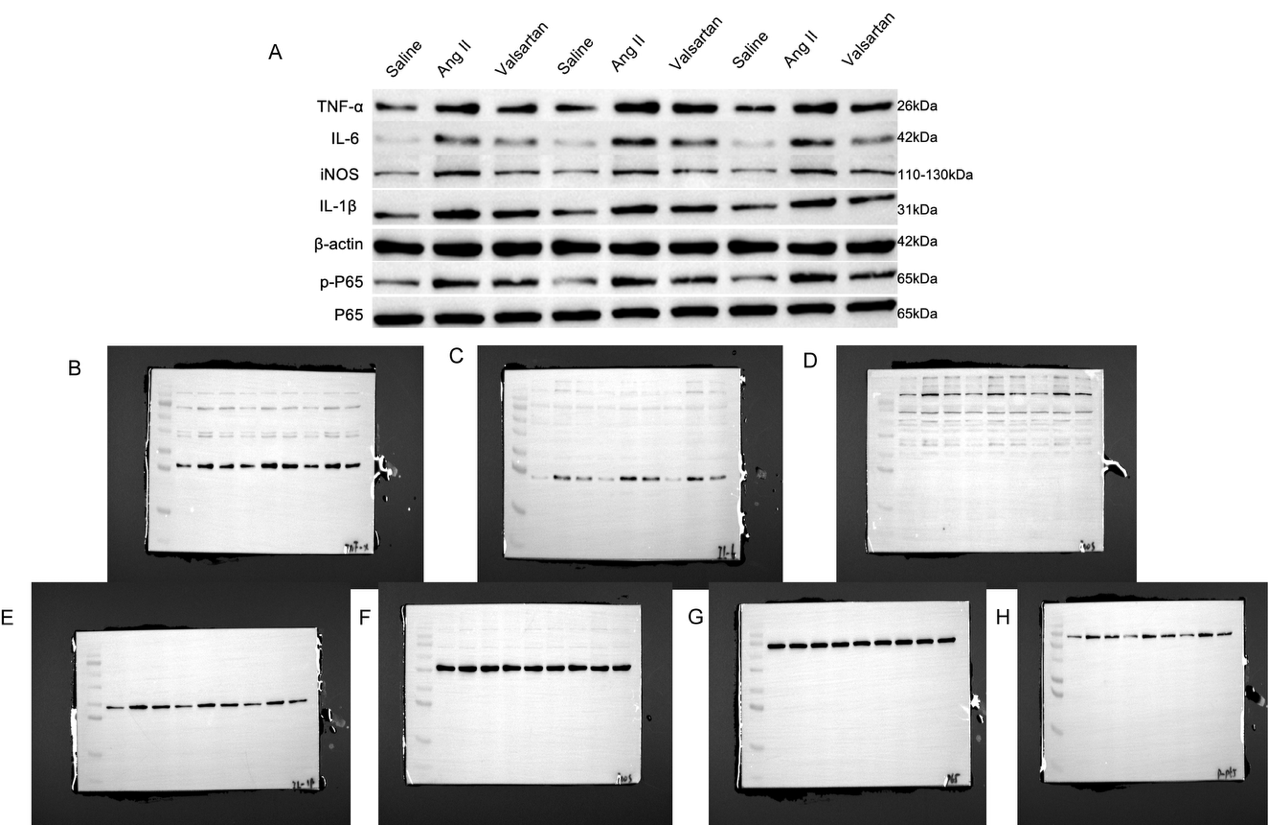


Figure S4. (A) WB strip of TNF-α, IL-6, iNOS, IL-1β and p-P65 with three repeats, respectively. (B-F) The original PVDF membrane of TNF-α, IL-6, iNOS, IL-1β and the relevant β actin. (D-E) The original PVDF membrane of p-P65 and the relevant control P65.
